# A Fibrinogen Alpha Fragment Mitigates Chemotherapy-Induced *MLL* Rearrangements

**DOI:** 10.3389/fonc.2021.689063

**Published:** 2021-06-18

**Authors:** Julia Eberle, Rahel Stefanie Wiehe, Boris Gole, Liska Jule Mattis, Anja Palmer, Ludger Ständker, Wolf-Georg Forssmann, Jan Münch, J. Christof M. Gebhardt, Lisa Wiesmüller

**Affiliations:** ^1^ Department of Obstetrics and Gynecology, Ulm University, Ulm, Germany; ^2^ Department of Physics, Institute of Biophysics, Ulm University, Ulm, Germany; ^3^ Core Facility Functional Peptidomics, Ulm University Medical Center, Ulm, Germany; ^4^ Pharis Biotec GmbH and Peptide Research Group, Institute of Immunology and Rheumatology, Hannover Medical School, Hannover, Germany; ^5^ Institute of Molecular Virology, Ulm University Medical Center, Ulm, Germany

**Keywords:** bioactive peptide, doxorubicin, Endonuclease G, hematopoietic stem and progenitor cells, inflammatory signaling, mixed lineage leukemia

## Abstract

Rearrangements in the *Mixed Lineage Leukemia* breakpoint cluster region (*MLL*bcr) are frequently involved in therapy-induced leukemia, a severe side effect of anti-cancer therapies. Previous work unraveled Endonuclease G as the critical nuclease causing initial breakage in the *MLL*bcr in response to different types of chemotherapeutic treatment. To identify peptides protecting against therapy-induced leukemia, we screened a hemofiltrate-derived peptide library by use of an enhanced green fluorescent protein (EGFP)-based chromosomal reporter of *MLL*bcr rearrangements. Chromatographic purification of one active fraction and subsequent mass spectrometry allowed to isolate a C-terminal 27-mer of fibrinogen α encompassing amino acids 603 to 629. The chemically synthesized peptide, termed Fα27, inhibited *MLL*bcr rearrangements in immortalized hematopoietic cells following treatment with the cytostatics etoposide or doxorubicin. We also provide evidence for protection of primary human hematopoietic stem and progenitor cells from therapy-induced *MLL*bcr breakage. Of note, fibrinogen has been described to activate toll-like receptor 4 (TLR4). Dissecting the Fα27 mode-of action revealed association of the peptide with TLR4 in an antagonistic fashion affecting downstream NFκB signaling and pro-inflammatory cytokine production. In conclusion, we identified a hemofiltrate-derived peptide inhibitor of the genome destabilizing events causing secondary leukemia in patients undergoing chemotherapy.

## Introduction

Chromosomal translocations, inversions, amplifications and complex rearrangements at the 11q human genomic locus encoding the lysine methyl transferase 2 A gene (*KMT2A*), also known as mixed lineage leukemia gene (*MLL*), are a hallmark of acute lymphoblastic leukemia (ALL) in infants and of therapy-induced acute myeloid leukemia/myelodysplastic syndrome (AML/MDS) in adults (1–3). *MLL* abnormalities were reported to be detectable in up to 85% of infant ALL and approximately one third of therapy-induced AML cases (2, 4). Underscoring their impact, these chromosomal changes correlate with poor prognosis, resistance to treatment and reduced overall survival (5, 6). Infant ALL cases have causally been linked with exogenous sources such as exposure to carcinogenic dietary components during fetal hematopoiesis (7–9). Therapy-induced leukemias have been observed after treatments with various cytostatics ranging from nucleoside analogs to alkylating agents, with a particularly short latency period after chemotherapies with anti-topoisomerase II inhibitory compounds like the epipodophyllotoxin etoposide or DNA intercalating drugs like the anthracycline doxorubicin (10–14). Providing further support for a mechanistic connection between infant ALL and therapy-induced AML, the vast majority of aberrations fall within a 0.4 kb breakpoint cluster region hotspot (*MLL*bcr) that is distinct from the cluster of *MLL* rearrangements in adult *de novo* ALL [reviewed in (2, 15)]. The *MLL*bcr sequence has been predicted to adopt complex secondary structures with hairpins that are known to be highly enriched at translocation breakpoints in human cancer genomes (2, 16). Such non-B DNA structures represent obstacles to the transcription and DNA replication machinery and are therefore subject to incisions by nucleases. *MLL*bcr rearrangements can be explained by error-prone repair attempts in apoptosis-escaping cells and can be recapitulated *ex vivo* in cultured lymphocytes as well as hematopoietic stem and progenitor cells (HSPCs) after exposure to nutritional risk factors or cytostatic drugs (8, 9, 17–19).

In our previous work we unraveled a therapy-induced replication stress-signaling pathway, which triggers nuclear accumulation of Endonuclease G (EndoG) and specific *MLL*bcr cleavage, whereby EndoG is recruited by the base excision repair machinery in an attempt to resolve stalled replication forks. Importantly, we showed that in cycling cells *MLL*bcr cleavage by EndoG is necessary for misrepair resulting in *MLL*bcr rearrangements to occur (19, 20). Of note, HSPCs, the leukemia cells-of-origin, are forced to enter the cell cycle upon stress stimuli such as transplantation, infection or chemotherapy (15). Replication stress may therefore represent the integrating signal triggering *MLL*bcr cleavage and consequently treatment-induced AML in patients treated with various types of cytostatics (2).

Etoposide and doxorubicin are widely applied in anti-cancer treatments showing efficacy against solid tumors, leukemias and lymphomas, including bladder, lung, breast and thyroid cancers, Hodgkin’s lymphoma, multiple myeloma and others (11, 21). However, their clinical use and their dosage are limited by adverse effects, mostly cardio- and hematological toxicities as well as treatment-induced leukemia. Etoposide is a topoisomerase II inhibitor known to cause *MLL* breakage (22). In our previous work we found EndoG to be involved in *MLL*bcr cleavage after etoposide treatment (20). Anthracyclins like doxorubicin exert multiple biological activities inducing DNA damage *via* DNA intercalation and generation of reactive oxygen species (ROS) and have as well been linked with *MLL* rearrangements in therapy-induced leukemia (23–25). In search of cytoprotective compounds, Jang and colleagues (26) screened and found two small molecule EndoG inhibitors. However, indiscriminate loss of all EndoG functions is not desirable given its importance in the homeostasis of mitochondria (27), an assumption which is supported by the cardiotoxic effects observed in knockout mice (28).

Given that the human peptidome is a large unexplored source of bioactive peptides with unknown functions that may be involved in *MLL*bcr rearrangements, we chose a screening approach to identify endogenous, hemofiltrate-derived peptides that mitigate these rearrangements during etoposide and doxorubicin treatments. Employing EGFP-based reporter cells we discovered such a peptide, namely a C-terminal 27-mer cleavage product of fibrinogen α, and discovered that it antagonizes toll-like receptor 4 (TLR4) - NFκB signaling. Our work provides evidence that inflammatory signaling plays a key modulatory role in one of the most severe side effects of cytostatic treatments. We further provide proof-of-concept that a peptide can block chemotherapy-induced localization of EndoG in the nucleus and therefore *MLL*bcr rearrangements by manipulating this pathway from the cellular surface.

## Material and Methods

### Cell Culture

Human WTK1 (29) and K562 (#ATCC CCL 243, American Type Cell Culture Collection) as well as the reporter cell line derivatives WTK1MLL and K562MLL, both stably transfected with the DNA recombination substrate pHR-EGFP/3’EGFP-MLLbcr.fwd (30), were cultured in RPMI 1640 (Gibco/Thermo Fisher Scientific, Waltham, Massachusetts, USA) supplemented with 10% fetal bovine serum (Biochrom, Berlin, Germany) and 5% L-Glutamine (Gibco/Thermo Fisher Scientific, Waltham, Massachusetts, USA). For serum-free experiments AIM V™ Medium (Gibco/Thermo Fisher Scientific, Waltham, Massachusetts, USA) was used. The HEK-Blue™ hTLR4 cells obtained by co-transfection with TLR4, MD-2 and CD14 co-receptor genes and an inducible SEAP (secreted embryonic alkaline phosphatase) reporter gene (InvivoGen, San Diego, California, USA) were cultured in DMEM without glucose, glutamine and phenol red (Gibco/Thermo Fisher Scientific, Waltham, Massachusetts, USA), which was supplemented with 4.5 g/l glucose (Sigma-Aldrich/Merck, St. Louis, Missouri, USA), 2 mM L-glutamine (Gibco/Thermo Fisher Scientific), 10% fetal bovine serum (Biochrom, Berlin, Germany), 1x penicillin-streptomycin (Gibco/Thermo Fisher Scientific, Waltham, Massachusetts, USA), 100 µg/ml Normicin™ (InvivoGen, San Diego, California, USA) and 1x HEK-Blue™ selection (InvivoGen, San Diego, California, USA). HeLa cells (ECACC, UK, London, 93021013) were cultured in DMEM (Gibco/Thermo Fisher Scientific, Waltham, Massachusetts, USA) including 10% FBS (Sigma-Aldrich/Merck, St. Louis, Missouri, USA), 1% sodium pyruvate (Gibco/Thermo Fisher Scientific, Waltham, Massachusetts, USA), 1% non-essential amino acids (Gibco/Thermo Fisher Scientific, Waltham, Massachusetts, USA) and 1% GlutaMAX (Gibco/Thermo Fisher Scientific, Waltham, Massachusetts, USA).

Human hemofiltrate was collected and analyzed considering ethical vote #91/17 (University of Ulm) and with the patient’s written informed consent prior to inclusion in the study. Umbilical cord blood samples were collected in the Department of Obstetrics and Gynecology of Ulm University and informed consent was obtained from mothers within 24 h after birth. Blood sample collections were approved by the local advisory board (approval #155/13, University Ulm). Hematopoietic stem and progenitor cells (HSPCs) were isolated by Melanie Rall-Scharpf and Heike Schreier.

Culturing was performed in a humid 5% CO_2_ incubator at 37°C. All cell lines were tested negative for mycoplasma by PCR.

### Treatment and Measurement of *MLL*bcr Rearrangements *via* FACS Analysis

K562MLL or WTK1MLL cells (1-2 x10^6^ cells in a final volume of 2 ml) were seeded in RPMI 1640 or AIM V™ medium and subjected to serial treatments according to the experimental procedure described in the legend of each figure. In the experiments with continuous treatment, a 4 h pre-treatment with Fα27 was followed by an exposure to peptide plus 10 µM etoposide for 72 h (Sigma-Aldrich/Merck, St. Louis, Missouri, USA) or doxorubicin (University Hospital Ulm, Germany) at the approximate IC50 dose (WTK1MLL: 0.5 µM). In the experiments with release of WTK1MLL cells from drug treatments, the 4 h incubation with Fα27 peptide was followed by another 4 h incubation period with peptide plus 0.5 µM doxorubicin. Subsequently, cells were washed with 2 ml of PBS and seeded in 2 ml of fresh AIM V™ medium without peptide and cytostatic drug followed by 72 h of cultivation. For the determination of recombination frequencies adjacent to the *MLL*bcr (*MLL*bcr rearrangements) the medium was removed and the cells were resuspended in PBS with 0.2% EDTA. The fractions of EGFP-positive cells among living cells were measured flow cytometrically by use of a FACSCalibur™ (BD Biosciences, San Jose, California, USA), whereby living cells were selected by the FSC/SSC gate and green fluorescent cells therein detected in the diagonal FL1/FL2 dot plot gate (31, 32). Autofluorescence of doxorubicin led to a fluorescence signal shift of the cells during FACS analysis requiring gate adjustment by a treated control without EGFP-reporter. To correct for inter-experimental variation mean recombination frequencies in peptide-free etoposide/doxorubicin controls were set to 100% in each experiment and relative percentages calculated for each single value. Peptide concentrations were chosen according to concentration-dependent responses of the different cell types during recombination measurements. Thus, WTK1MLL cells were exposed to 1 mg/ml Fα27 for etoposide and 10 µg/ml Fα27 for doxorubicin treatments to reach down-regulation of *MLL*bcr rearrangements by at least 40%.

For the experiments engaging disulfiram (Sigma-Aldrich/Merck, St. Louis, Missouri, USA) or *N*-acetyl cysteine (NAC) (Sigma-Aldrich/Merck, St. Louis, Missouri, USA) additional pre-incubation times of 5 h for disulfiram or 1 h for NAC were added to the protocol, i.e. before the peptide incubation period, whereby disulfiram or NAC were kept in the media as long as peptide and cytostatic treatments took place.

### Human Hemofiltrate-Derived Peptide Screen

A peptide library was generated starting from hemofiltrate of patients suffering from chronic renal failure as previously described (33). Briefly, blood was filtered across a membrane with a cut-off of 20-30 kDa. Peptides in the filtrate were separated by cation-exchange chromatography resulting in eight eluates which were then separated by reverse phase HPLC into 48 fractions each (34). First round screening of the resulting 384 fractions was performed in K562MLL cells with a chromosomally integrated EGFP-based reporter construct for *MLL*bcr rearrangements (30). Cells were seeded in 2 ml culture medium and pre-treated for 4 h with 90 µl of each fraction corresponding to 5 l of hemofiltrate. Subsequently, the chemotherapeutic etoposide (10 µM) was included and cells cultivated for 72 h until recombination measurements by FACS. Fractions inducing statistically significant decreases in mean *MLL*bcr recombination frequencies were subjected to second round screening after re-chromatography by analytical reversed phase chromatography. Second round screening of fraction E8F08 revealed fraction 11 with the most pronounced downregulation of etoposide-induced recombination and was subjected to MALDI mass spectrometry analysis and Edman Sequencing. For MALDI-MS samples were re-suspended in a MALDI matrix solution containing alpha-cyano-4-hydroxy cinnamic acid (HCCA, 8 mg/ml, Fluka, Morristown, USA) with 6-desoxy-L-galactose additive in 50/49/1 (v/v) 0.2% TFA/acetonitrile/acetone. Then, 0.5 µl of the sample/matrix mixture were spotted on a target and spectra were acquired in linear mode (Applied Biosystems 4700 Proteomics Analyzer MALDI-TOF/TOF). Sequencing was carried out by conventional Edman degradation detecting the phenyl isothiocyanate labeled amino acids (33, 34).

### Peptide Synthesis and Purification

The peptides were synthesized using standardized Fmoc chemistry by solid-phase peptide synthesis (SPPS) on an automated peptide synthesizer (Liberty blue, CEM, Kamp-Lintfort, Germany). An amino acid with a protecting group was added to the first amino acid, which was linked to the resin. After activation the subsequent amino acid was coupled to grow the peptide chain in cycles. Finally, the full-length peptide was removed from all protecting groups and the solid-phase resin. The peptide was purified with a HPLC-1100 and characterized *via* mass spectrometry. The crude peptide was stored at 4°C and after solubilization at -20°C. The sequences of the used peptides were Fα27 (MADEAGSEADHEGTHSTKRGHAKSRPV), Scramble 1 (LASCEAYIKRGNVHTDPGLGASVLQAF), Scramble 2 (RSDDEHASKHHVTPEEAGRMSATAGSK), Fα27-Flag (MADEAGSEADHEGTHSTKRGHAKSRPVGSSGSSDYKDDDDK) and Flag-Fα27 (DYKDDDDKGSSGSSMADEAGSEADHEGTHSTKRGHAKSRPV). 10 µg/ml Fα27, Scramble 1 and Scramble 2 correspond to 3.5 µM, 15 µg/ml Fα27-Flag and Flag-Fα27 correspond to 3.5 µM.

### Cell Cycle Analysis

Cell cycle analysis was performed as detailed in the **Supplement**.

### DNA Double-Strand Break (DSB) Repair Analysis *via* FACS

WTK1 or WTK1MLL cells were seeded at a density of 4 x10^6^ in AIM V™ medium and treated with 1 mg/ml Fα27 for 4 h. After the pre-incubation period cells were washed with RPMI 1640 Medium, with neither glutamine nor phenol red (Gibco, Thermo Fisher Scientific) and resuspended in 400 µl of this medium. For comparative analysis of extrachromosomal DSB repair activities 10 µg of pCMV-I-SceI plasmid expressing the rare-cutting meganuclease I-*Sce*I and 10 µg of the plasmid EJ-EGFP for determination of MMEJ or of HR-EGFP/3’EGFP for homologous repair (35) were added to the WTK1 cells. For analysis of chromosomal homologous repair, the stable reporter WTK1MLL cells were electroporated with 10 µg plasmid expressing I-*Sce*I only. Mixtures of plasmids and cells were transferred in a Gene Pulser^®^/Micropulser™ electroporation cuvette 4 mm (Bio-Rad Laboratories, Hercules, California, USA) and pulsed at 200 V and 1050 µF with the Gene Pulser Xcell™ electroporation system (Bio-Rad Laboratories, Hercules, California, USA). The cells were added to AIM V™ Medium and the Fα27 concentration adjusted. For the determination of DSB repair frequencies the medium was removed, the cells were resuspended in PBS/0.2% EDTA and the fraction of green fluorescent cells among living cells (FSC/SSC gate) determined with a FACSCalibur™ (BD Biosciences, San Jose, California, USA) using the diagonal gating method in the FL-1/FL-2 dot plot (35). Living EGFP-positive cell fractions in extrachromosomal DSB repair assays were normalized to the transfection efficiencies to calculate the DSB repair frequency. Mean recombination frequencies in peptide-free controls were set to 100% in each experiment and relative percentages calculated.

### Bulk and Single Molecule Imaging of Fα27

One day before the measurement, HeLa cells were grown at 37°C and 5% CO_2_ in DMEM medium (Gibco/Thermo Fisher Scientific, Waltham, Massachusetts, USA) including 10% charcoal-stripped FBS (Gibco/Thermo Fisher Scientific, Waltham, Massachusetts, USA), 1% sodium pyruvate (Gibco/Thermo Fisher Scientific, Waltham, Massachusetts, USA), 1% non-essential amino acids (Gibco/Thermo Fisher Scientific, Waltham, Massachusetts, USA) and 1% GlutaMAX (Gibco/Thermo Fisher Scientific, Waltham, Massachusetts, USA) in 35 mm glass bottom μ-dishes (Ibidi, Gräfelfing, Germany). Before the measurement, we added TAMRA-Fα27 (Pepscan, RC Lelystad, The Netherlands) in a final concentration of 0.27 µM in OptiMEM (Gibco/Thermo Fisher Scientific, Waltham, Massachusetts, USA) and incubated the cells for 4 h at 37°C and 5% CO_2_. Afterwards, we imaged the cells on a custom built spinning disk microscope (36) using a 532 nm laser to visualize TAMRA-Fα27.

For single molecule imaging of TAMRA-Fα27, we followed the same procedure, but grew cells on Delta-T glass bottom dishes (Bioptechs Inc., Butler, Pennsylvania, USA). The final concentration of TAMRA-Fα27 was 2.7 nM. We imaged single TAMRA-Fα27 molecules using HILO microscopy (36) with a 561 nm laser at 1 kW/cm^2^ and 10 ms acquisition time.

### Immunofluorescence Microscopy

After a 4 h treatment with Fα27 and 4 h with Fα27 plus cytostatic at the indicated concentrations the cells were washed with PBS and spun on microscopic slides coated with Poly-L-Lysine (Sigma-Aldrich/Merck, St. Louis, Missouri, USA). Then, they were pre-extracted with 0.2% Triton/PBS for 30 s and washed for 5 min with PBS. For fixation the slides were incubated for 10 min in 3.7% formaldehyde and washed with PBS three times. Subsequently, cells were permeabilized with 0.5% Triton/PBS for 12 min and PBS washed three times followed by blocking with 5% goat serum/PBS for 1 h. For immunostainings the primary antibodies directed against γH2AX (Merck Millipore, Burlingtion, Massachusetts, USA 05-636) or EndoG (Santa Cruz Biotechnology, Dallas Texas, USA, sc-365359) were applied 1:1000 in 5% goat serum/PBS for 1 h at 37°C. Incubation with the secondary antibody Alexa Fluor^®^ 488 (Thermo Fisher Scientific, Waltham, Massachusetts, USA, A11001) at 1:1000 in 5% goat serum/PBS took place for 45-60 min at 37°C followed by washing and mounting with VectaShield Mounting Medium for Fluorescence with Dapi (Vector Laboratories, Burlingame, California, USA) and sealed under cover slips. The slides were subjected to high content imaging with a BZ-9000 microscope (Keyence, Neu-Isenburg, Germany), objective 100x/1.45 oil (Nikon, Tokio, Japan) and automated analysis of immunostained foci in DAPI-stained nuclei with BZ-II Analyzer software (Keyence, Neu-Isenburg, Germany). To ensure detection of specific signals negative controls without primary antibodies were included and the same exposure times as well as intensity and minimal focus size thresholds kept throughout each experimental set.

### Proximity-Ligation-Assay (PLA)

HEK-Blue™ hTLR4 cells were seeded on cover slips coated with sterile Poly-L-Lysine (Sigma-Aldrich/Merck, St. Louis, Missouri, USA) for 72 h. Then, the cells were washed with PBS and treated for 2 h with H_2_O, Flag-Fα27 or Fα27-Flag in two different concentrations 15.09 µg/ml and 150.9 µg/ml corresponding to 10 µg/ml Fα27 and 100 µg/ml Fα27, i.e. 3.5 µM and 35 µM peptides, respectively. After treatment the cells were washed for 5 min with PBS and fixed for 10 min in 3.7% formaldehyde. The PLA staining was performed following the manufacturer´s instructions (Sigma-Aldrich/Merck, St. Louis, Missouri, USA) using Duolink™ In Situ Detection Reagent Orange, Duolink™ In Situ PLA Probe Anti-Rabbit PLUS, Duolink™ In Situ PLA Probe Anti-Mouse MINUS and Duolink™ In Situ Mounting Medium with DAPI. The primary antibodies TLR4 (Santa Cruz Biotechnology, Dallas Texas, USA, sc-293072), Anti-DDDDK tag/Flag tag (Abcam, Cambridge, UK, ab205606) as well as for positive controls Ku80 (Thermo Fisher Scientific, Waltham, Massachusetts, USA, S.669.4), Ku70 (Abcam, Cambridge, UK, ab202022) and MD2 (Abcam, Cambridge, UK, ab24182) were incubated overnight at 4°C. Image analysis was performed as described for immunofluorescence microscopy. To ensure detection of specific signals negative controls with TLR4 or Flag antibody only or PLA without any antibody were performed. Minimal focus size thresholds and foci size were set independently for each experiment and kept throughout each experimental set.

### Chromatin Immunoprecipitation (ChIP)

After a 4 h treatment with Fα27 and 4 h with Fα27 plus cytostatic the cells were fixed for 10 min in 1% formaldehyde in the medium on a shaker. Then, glycine was added to the medium to a final concentration of 138 mM and shaking continued for 5 min. Subsequently, cells were centrifuged at 4°C. All following steps were executed on ice or at 4°C using ice cold buffers. The cells were washed with PBS, centrifuged, resuspended in PBS, transferred to a 1.5 ml reaction tube, re-centrifuged, the supernatant removed and the cells shock-frozen in liquid nitrogen and stored at -80°C for further use. Cell pellets were dispensed in 1 ml cell lysis buffer (5.0 mM PIPES, pH 8.0; 85 mM KCl; 0.5% NP-40) with 0.5 mM PMSF (Sigma-Aldrich/Merck, St. Louis, Missouri, USA) and 0.5% of Protease Inhibitor Cocktail (PIC) (Sigma-Aldrich/Merck, St. Louis, Missouri, USA). Then the cells were dounced for 5 min (Wheaton/DWK Life Sciences, Wertheim, Germany), centrifuged, resuspended in 1 ml nuclei lysis buffer (50 mM Tris-HCl, pH 8.0; 10 mM EDTA; 1.0% SDS), incubated for 10 min and sonicated 6 times for 10 s with intermittent 1 min breaks. The resulting suspension was frozen in liquid nitrogen and stored at -80°C for further use. Aliquots (50 µl) taken before freezing were diluted fourfold in pure water, NaCl added to a final concentration of 250 mM and samples incubated overnight at 65°C. Thereafter, 0.05 µg/µl RNaseA (Invitrogen/Thermo Fisher Scientific, Waltham, Massachusetts, USA) was added and the sample incubated for 15 min at 37°C followed by 0.025 µg/µl Proteinase K (Sigma-Aldrich/Merck, St. Louis, Missouri, USA) treatment for 90 min at 42°C. DNA and proteins were separated with phenol-chloroform extraction and the DNA precipitated with 0.2 M sodium acetate (pH 5.2) and 62.5% ethanol overnight at -20°C. DNA pellets were dried and resuspended in 30 µl TE buffer (10 mM Tris-HCL, pH 8.0; 1 mM EDTA). These samples were used for input DNA assessment. The remaining cell suspensions were thawed on ice, centrifuged and volumes containing 2-20 µg DNA diluted in a total volume of 300 µl dilution buffer (16.7 mM Tris-HCl, pH 8.0; 167 mM NaCl; 1.2 mM EDTA; 0.01% SDS; 1.1% Triton X-100 including 0.5 mM PMSF and 0.5% PIC). For pre-clearing protein G sepharose salmon sperm DNA beads were added, i.e. Protein G Sepharose™ 4 Fast Flow (GE Healthcare, Chicago, Illinois, USA) in 50 µl TE-buffer supplemented with 1 mg/ml BSA and 0.05% sodium azide including 0.4 mg/ml Sheared Salmon Sperm DNA (Ambion^®^ by life technologies™/Thermo Fisher Scientific, Waltham, Massachusetts, USA). Thereafter samples were rotated for 1-2 h. Following centrifugation, pre-cleared supernatants were transferred to new reaction tubes and incubated together with the antibody of interest: γH2AX, Merck Millipore, Burlington, Massachusetts, USA, 05-636; EndoG, Santa Cruz Biotechnology, Dallas Texas, USA, sc-365359; mouse IgG, Santa Cruz Biotechnology, Dallas Texas, USA, sc-2025. After rotation overnight 50 µl of protein G-agarose-salmon sperm DNA beads were added and rotation continued for 2 h. Samples were centrifuged and bead pellets washed four times at room temperature by 10 min rotation and centrifugation with high-salt wash buffer (50 mM HEPES, pH 7.9; 500 mM NaCl; 1.0 mM EDTA; 0.1% SDS; 1.0% Triton X-100; 0.1% deoxycholate) and twice with TE. Beads were resuspended in 300 µl elution buffer (50 mM Tris-HCl, pH 8.0; 10 mM EDTA; 1.0% SDS) and incubated 2 h at 55°C with 0.0625 µg/µl proteinase K. Afterwards, samples were incubated overnight at 65°C, then centrifuged and supernatants transferred into new reaction tubes. The DNA was isolated as described before and amplified by PCR. The PCR products were separated by agarose gel electrophoresis (2.5%) and visualized by SYBR Save DNA gel stain (Thermo Fisher Scientific, Waltham, Massachusetts, USA). The primer (Biomers.net, Ulm, Germany) sequences were as follows: For amplification of the HR-EGFP/MLLbcr fragment fwd primer 5´-ACCACTACCAGCAGAACACC-3´ and rev primer 5´- ATACGAAACAGTTGTAAGTATCGTC-3´; for HR-EGFP/MLLbcr nested PCR fwd primer 5´-ACAACCACTACCTGAGCACC-3´ and rev primer 5´-GCTTGATATCGAATTCCTGCAGCC-3´; for GAPDH fwd primer 5´-CCCAACTTTCCCGCCTCTC-3´ and rev primer 5´-CAGCCGCCTGGTTCAACTG-3´.

### qRT-PCR

WTK1MLL cells were treated as described for the analysis of *MLL*bcr rearrangements *via* FACS. Total RNA isolation was done with the RNeasy Plus Mini Kit (Qiagen, Hilden, Germany) following the manufacturer´s instructions. Reverse transcription was performed using the QuantiTec Reverse Transcription Kit (Qiagen, Hilden, Germany) following the instructions for 1 µg RNA. For qPCR 3.3 µl cDNA (1:20 diluted) were mixed with 5 µl SensiFAST Probe LO-ROX mix (2x) from the SensiFAST Probe LO-ROX Kit (Bioline, London, UK), 0.5 µl sensor primer and 1.2 µl H_2_O. The PCR was performed on the qTower (Analytik Jena, Jena, Germany) by using the program 2 min 95°C and 40 cycles of 10 s 95°C and 20 s of 60°C. Controls were ACTB VIC (Thermo Fisher Scientific, Waltham, Massachusetts, USA), HSP90 VIC (Thermo Fisher Scientific, Waltham, Massachusetts, USA),YWHAZ VIC (Thermo Fisher Scientific, Waltham, Massachusetts, USA), ACTB HEX (Bio-Rad Laboratories, Hercules, California, USA) and YWHAZ HEX (Bio-Rad Laboratories, Hercules, California, USA). The genes of interest were analyzed with IFN-gamma Fam, IL-6 Fam, IL-8 Fam and TLR4 Fam (Bio-Rad Laboratories, Hercules, California, USA).

### HEK-Blue™-Assay

For the HEK-Blue™-Assay the HEK-Blue™ hTLR4 cells were detached and resuspended in HEK-Blue™ Detection (InvivoGen, San Diego, California, USA) at a density of 140000 cells/ml. The peptides were diluted in PBS and 15 µl of the solutions added to the wells to reach the indicated concentrations in the final volume of 200 µl. Subsequently, 180 µl of the cell suspensions were added for 2 h. Thereafter, cells were treated with final concentrations in 200 µl of 10 ng/ml LPS (InvivoGen, San Diego, California, USA) for 10 h or 0.16 µM doxorubicin (IC80 for 24 h treatment) for 4 h diluted in a volume of 5 µl. Then, doxorubicin treated cells were released into fresh medium. For the release, the cells were centrifuged, the supernatant removed and cells resuspended in 100 µl 1xPBS. After a second centrifugation step the supernatant was removed and 20 µl 1xPBS and 180 µl HEK-Blue™ Detection added for 72 h. Finally, optical densities were measured at 655 nm.

### Breakage PCR of the *MLL*bcr in HSPCs

Human stem and progenitor cells (HSPCs) were isolated from cord blood essentially as described in Kraft et al. (18). Cells were thawed at 37°C. One cell vial volume equivalent to RPMI 1640 (Gibco/Thermo Fisher Scientific, Waltham, Massachusetts, USA) with 10% fetal bovine serum (Biochrom, Berlin, Germany) was slowly dropped to the cell suspension. After an incubation time of 1 min another volume equivalent to the cell mixture with medium was added to the cells by slowly dropping. This step was repeated three times. After centrifugation, the cells were suspended in StemSpan™ SFEM (Stemcell Technologies, Vancouver, Canada) with 1% penicillin-streptomycin (Gibco/Thermo Fisher Scientific, Waltham, Massachusetts, USA) and 1% StemSpan™ CC100 (Stemcell Technologies, Vancouver, Canada). HSPCs were cultured at a density of 0.75x10^6^ cells/ml for 72 h. For the experiment the cells were seeded in fresh culture medium, treated for 2 h with 10 µg/ml Fα27 followed by 4 h treatment with the IC90 dose of doxorubicin (0.072 µM) pre-established in WTK1 cells. Genomic DNA was isolated with the QIAamp DNA Mini Kit (Qiagen, Hilden, Germany) following the manufacturer´s advices and amplified *via* PCR. The PCR products were separated by agarose gel electrophoresis (2.5%) and visualized by SYBR Save DNA gel stain (Thermo Fisher Scientific, Waltham, Massachusetts, USA). The primer (Biomers.net, Ulm, Germany) sequences were as follows:

MLL (Set2), fwd 5´-TTGGGTGTAATCAGTTGCCTATT-3´ and rev 5´-GGGTGATAGCTGTTTCGGCA-3´

MLL (Intron20), fwd 5´-GCAACACAGGGCCCTAGTTAAT-3´ and rev 5´-AGGCAAATCAGTCACCTTTTAATCA-3´.

### Statistical Analysis

All experiments were repeated independently at least twice. P values for unpaired, nonparametric data were determined using Mann-Whitney-U test (two-tailed) in case of statistical significance with the Kruskal-Wallis H-test. When analyzing statistical significance of data after normalization to 100% (paired, nonparametric), p values were calculated using Wilcoxon-signed ranks test in case of statistical significance with the Friedman test. All calculations were done with GraphPad 8 (GraphPad Software, San Diego, California, USA). During the library screen statistical significances of differences between mean recombination frequencies were calculated using χ^2^ test for the initial testing (n=2) and two-tailed Mann-Whitney-U test after additional values were obtained (n=4).

## Results

### Identification of Hemofiltrate-Derived Peptide Fα27 That Mitigates Chemotherapy-Induced Rearrangements Underlying Development of Secondary Leukemia

**Figure 1:**
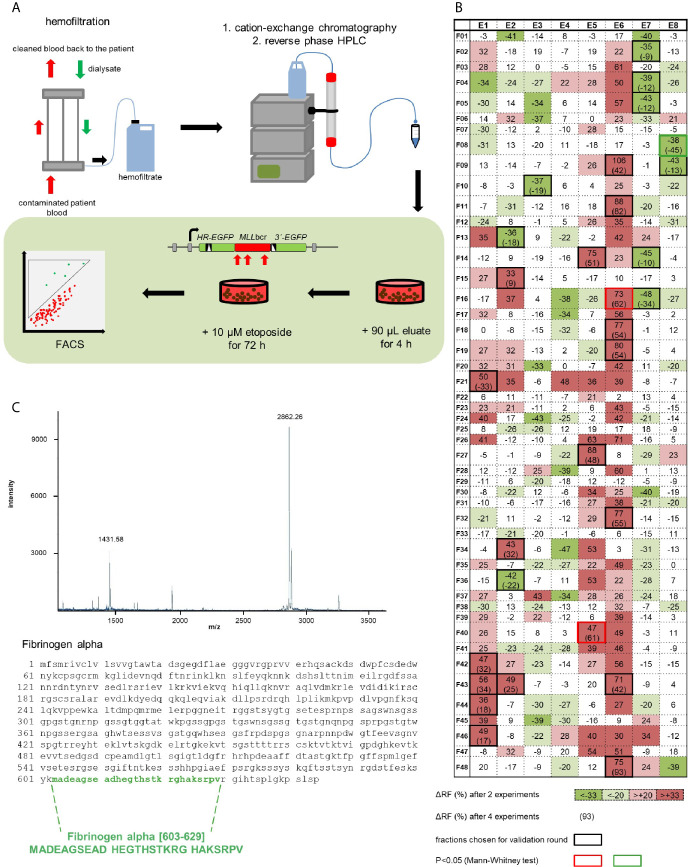
Identification of a human peptide from hemofiltrate reducing etoposide-induced rearrangements at the *MLL*bcr. **(A)** Principle of the peptide screen. Hemofiltrate was subjected to cation exchange chromatography and subsequent reversed phase high-performance liquid chromatography (HPLC) resulting in 384 eluate fractions, of which an aliquot of 90 µl each was added to a 2 ml culture of the cell line K562MLL with chromosomally integrated EGFP-based reporter construct for *MLL*bcr rearrangements. After a pre-treatment for 4 h the chemotherapeutic drug etoposide (10 µM) was included for 72 h. *EGFP* gene reconstitution was triggered by etoposide-induced *MLL*bcr breakage and recombination between two differentially mutated *EGFP* genes adjacent to the *MLL*bcr. *MLL*bcr rearrangements were thus measured as recombination frequencies determining the fraction of green fluorescent cells compared to the total population of live cells (FSC/SSC gate) by FACS analysis using the diagonal FL1-FL2 Dot Plot gate. **(B)** First round screening pattern. Relative changes of etoposide-induced recombination with versus without preincubation of cells with eluate are indicated for each HPLC fraction in %. Statistical significances of differences between mean recombination frequencies from two independent experiments according to χ^2^ test are visualized by gradual color changes, whereby red indicates increased and green decreased etoposide-induced recombination. For fractions with mean difference (Δ) ≥33% (p< 0.05 or SD<Δ) validation experiments were performed (black frames) resulting in n=4 (red or green frame: p<0.05 according to Mann-Whitney U test). **(C)** Identification of Fibrinogen alpha [603-629] by mass spectrometry (MALDI-MS) and Edman analysis of fraction E8F08 after HPLC purification. The MALDI-MS spectrum shows the single and the double charged peptide with a molecular weight of 2862 Da representing a C-terminal portion of fibrinogen α. See also **Figure S1**.

To identify a human peptide which protects against leukemic *MLL*bcr rearrangements during chemotherapeutic treatment, we screened a hemofiltrate-derived library from patients suffering from renal failure (33, 37). During hemofiltration, patients’ blood was filtered through a membrane with a 20-30 kDa cut-off and the remaining peptides and small proteins were separated by cation exchange and reverse-phase high-performance liquid chromatography (HPLC) as outlined in **Figure 1A** (33). For functional analysis of the resulting 384 fractions (eight pH pool eluates each containing 48 fractions), cells carrying a chromosomally integrated EGFP-based reporter construct for *MLL*bcr rearrangements (30) were pre-treated with each eluate for 4 h and subsequently exposed to the chemotherapeutic drug etoposide (10 µM) for 72 h. Etoposide treatment conditions were previously established and demonstrated to induce a one order of magnitude increase of *MLL*bcr rearrangements in K562MLL and WTK1MLL reporter cells derived from human erythroleukemia and human lymphoblastoid cells, respectively (30). Initially, two values (recombination frequencies determined by FACS) were obtained with each fraction. The 45 fractions that showed significant differences in recombination frequency by χ^2^ test were used to obtain additional measurements enabling more strict statistical analysis. This identified three fractions inducing statistically significant changes, namely E5F40 as well as E6F16 with stimulatory and, most interestingly, E8F08 with inhibitory effects protecting against *MLL*bcr rearrangements (**Figure 1B**). DNA content analysis revealed small changes in the cell cycle distribution or apoptotic cell death upon pre-incubation with E8F08 (**Figure S1A**). Because of our interest in a decrease of recombination we proceeded with fraction E8F08 and subjected it to re-chromatography by reverse-phase chromatography. Second round screening of the resulting 44 fractions identified fraction 11 with the most pronounced decrease in recombination (**Figure S1B**). MALDI mass spectrometry analysis and Edman Sequencing identified a highly purified peptide in the protective fraction 11 with a molecular mass of 2,862 daltons and a theoretical isoelectric point (pI) of 6.23, encompassing the 27 amino acids 603 to 629 of the fibrinogen alpha chain, i.e. amino acids MADEAGSEADHEGTHSTKRGHAKSRPV (Fα27) (**Figure 1C**).

### Fα27 Differentially Affects *MLL*bcr Rearrangements After Treatment With Etoposide and Doxorubicin

To examine the efficacy of chemically synthesized Fα27, we pre-incubated the reporter line K562MLL for 4 h with increasing peptide concentrations before exposure to chemotherapeutic drugs according to the treatment protocols established in our earlier investigation (30). Serum-free AIM V™ Medium, alleviating potential peptide proteolysis in FCS, improved the efficacy of the peptide, so that lower Fα27 concentrations were required for desired results and serum-free culture conditions were chosen for further experiments (data not shown). In this way we found that Fα27 reduced recombination frequencies in the chromosomally integrated *MLL*bcr reporter in K562MLL cells after etoposide (10 µM) or doxorubicin (2 µM) treatment with IC50 values for Fα27 of 15.2 μg/ml (5.3 μM) and 7.2 μg/ml (2.5 μM), respectively.

**Figure 2:**
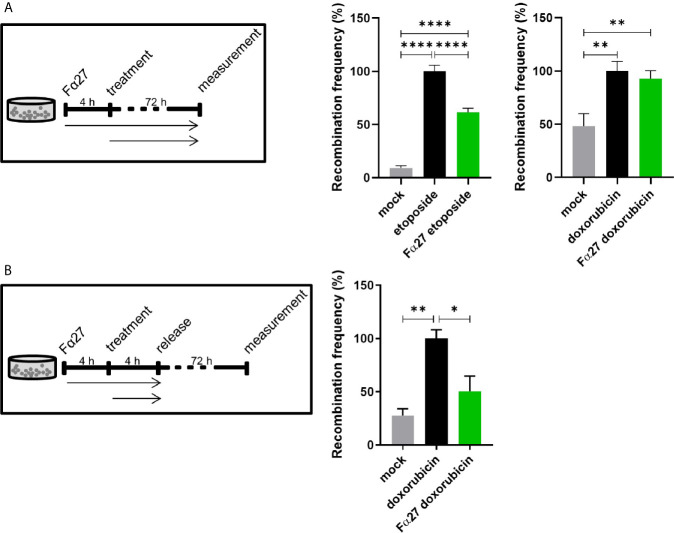
Fα27 differentially affects *MLL*bcr rearrangements in WTK1MLL cells after etoposide and doxorubicin treatment. **(A)** Continuous exposure of WTK1MLL cells to peptide and chemotherapeutic drugs. WTK1MLL cells were treated for 4 h with 1 mg/ml Fα27 followed by 72 h with Fα27 plus 10 µM etoposide or 10 µg/ml Fα27 followed by Fα27 plus 0.5 µM doxorubicin, respectively (n=12 obtained in four independent experiments). EGFP-positive cells were counted after 72 h by FACS. After etoposide but not doxorubicin treatment Fα27 reduced the recombination frequency significantly. **(B)** Release of WTK1MLL cells after peptide- and doxorubicin treatment. WTK1MLL cells were treated for 4 h with 10 µg/ml Fα27 followed by 4 h with Fα27 plus 0.5 µM doxorubicin treatment (n=6 from two individual experiments). Thereafter, the cells were released by washing with PBS and seeding in fresh medium. FACS analysis showed a significant reduction of the doxorubicin-induced recombination frequency by Fα27 pre-treatment. Mean recombination frequencies induced by etoposide (8.5x10^-5^) and by doxorubicin (1.8x10^-5^) were set to 100% for each experimental day. All values are represented as mean +SEM (*p < 0.05, **p < 0.01, ****p < 0.0001).

To exclude a mere cell line-dependent effect of Fα27 on chemotherapy-induced *MLL*bcr rearrangements, we examined another reporter line, namely WTK1MLL. Therefore, we pre-incubated the cells with Fα27 followed by combined treatment with Fα27 plus etoposide or doxorubicin. Fα27 significantly decreased etoposide-induced recombination at the *MLL*bcr by 38%, but did not alter recombination in the presence of doxorubicin (**Figure 2A**). While etoposide forms covalent topoisomerase poisoning complexes, doxorubicin intercalates into the double helix (38, 39). We therefore wondered whether doxorubicin interferes with the enzymatic machinery processing DNA lesions or participating in *EGFP* reconstitution within the *MLL*bcr reporter. Consequently, we modified our treatment protocol such that WTK1MLL cells were washed and released into fresh medium after sequential pre-incubations with Fα27 and Fα27 plus doxorubicin (**Figure 2B**). Under these conditions FACS measurements indicated a significant decrease of recombination by 50%.

To exclude potential confounding factors, we assessed survival by quantification of live cells in the FSC/SSC gate during FACS measurements of EGFP-positive cells in these live cell populations. **Figures S2A, B** reveal that cell death induced by etoposide or doxorubicin treatments was not influenced by Fα27 applied at concentrations of 1 mg/ml and 10 µg/ml, respectively. Additionally, we tested the specificity of the effect of Fα27 by comparison with two scrambled peptides (**Figure S2C**). Peptide Scramble 1 was randomly composed of amino acids reflecting the average frequency of occurrence, whereas peptide Scramble 2 consisted of the same amino acids as Fα27 in a different order. When compared to these two peptides Fα27 again caused reduced recombination after doxorubicin treatment, namely by 53% and 37%, providing evidence for a specific effect.

Altogether, we verified that chemically synthetized Fα27, but not its scrambled counterpart protected against chemotherapy-induced *MLL*bcr rearrangements in different cell types without affecting cell death. Interestingly, the *MLL*bcr protective effect differed between a treatment with the cytostatic drug etoposide or doxorubicin, suggesting an impact of the respective mode-of-action.

### Fα27 Has a Moderate Effect on DSB Repair That Can Partially Explain Protection From *MLL*bcr Rearrangements

**Figure 3:**
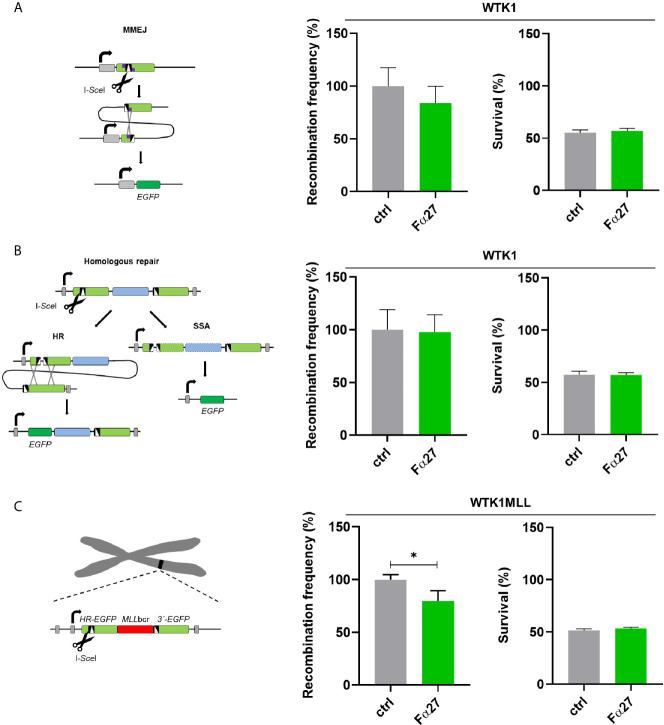
Influence of Fα27 on I-*Sce*I-induced MMEJ and homologous repair. **(A)** Scheme and results of MMEJ analysis following repair substrate cleavage by I-*Sce*I. WTK1 cells were pre-treated with 1 mg/ml Fα27 for 4 h and then transfected with two plasmids, namely the repair construct for MMEJ and the I-*Sce*I endonuclease expression plasmid (18). Targeted cleavage by I-*Sce*I splits the mutated EJ-EGFP gene, so that MMEJ repair of the reporter permits *EGFP* reconstitution *via* pairing of adjacent microhomologies. EGFP-positive cells were measured by FACS 48 h post-transfection (n=9 obtained in three individual experiments). Mean recombination frequencies of controls were set to 100% each (absolute mean: 2.9x10^-5^). **(B)** Scheme and results of homologous repair analysis following cleavage by I-*Sce*I. After cleavage by I-*Sce*I either HR or SSA can reconstitute an intact *EGFP* gene. A 4 h pre-treatment with 1 mg/ml Fα27 and transfection with homologous repair reporter as well as I-*Sce*I expression plasmid was followed by 48 h cultivation and FACS analysis (n=8-9 from three individual experiments). Mean recombination frequencies of controls were set to 100% each (absolute mean: 7.1x10^-4^). **(C)** Repair of I-*Sce*I-induced DSBs in the same chromosomally integrated construct also used for detection of drug-induced *MLL*bcr rearrangements. WTK1MLL cells were treated with 1 mg/ml Fα27 for 4h, transfected with I-*Sce*I expression plasmid and FACS analysis performed 48 h post-transfection (n=8-9 obtained in three independent experiments). Mean recombination frequencies of controls were set to 100% each (absolute mean: 1.2x10^-4^). All data represent mean +SEM (*p < 0.05). Corresponding survival data obtained by FACS analysis of live cells in the FSC/SSC plot are displayed in the right panel each.

DSBs are the predominating genotoxic lesions caused by etoposide, whereas doxorubicin induces a larger spectrum of DNA lesions (38, 39). To interrogate the possibility that Fα27 affects DSB repair, we engaged EGFP-based reporter constructs for the analysis of specific DSB repair pathways following targeted cleavage by the endonuclease I-*Sce*I. First, we measured microhomology-mediated end joining (MMEJ), and in a second set of experiments, homologous repair covering the pathways single-strand-annealing (SSA) and homologous recombination (HR) in WTK1 cells. For the analysis we pre-incubated the cells for 4 h with Fα27 and then introduced the repair constructs for MMEJ and homologous repair together with I-*Sce*I expression construct by electroporation. This was followed by cultivation in Fα27-containing medium in analogy to the experimental conditions established for treatment with etoposide, the drug mostly inducing DSBs. Both MMEJ and homologous repair did not show statistically significant differences after Fα27 treatment (**Figures 3A, B**). To examine DSB repair in the chromosomal context and in the same reporter construct that served to measure *MLLbcr* rearrangements, we expressed I-*Sce*I in WTK1MLL cells. The chromosomally integrated reporter comprises an I-*Sce*I recognition site next to the *MLLbcr*. Under these conditions Fα27 induced a 20% reduction of I-*Sce*I induced repair (**Figure 3C**). As seen before for chemotherapeutic drug exposures (**Figure S2**), the fraction of surviving cells was the same with and without Fα27 treatment (**Figure 3**, right panels) when targeted DSBs were introduced.

These results revealed that Fα27 downregulates the repair mechanism of DSBs to a lesser extent than *MLLbcr* rearrangements (**Figure 2**) suggesting that another component contributing to Fα27´s protective effect still had to be discovered.

### Focal Accumulation of EndoG in the Nucleus and Local Binding to the *MLL*bcr Is Decreased by Fα27

**Figure 4:**
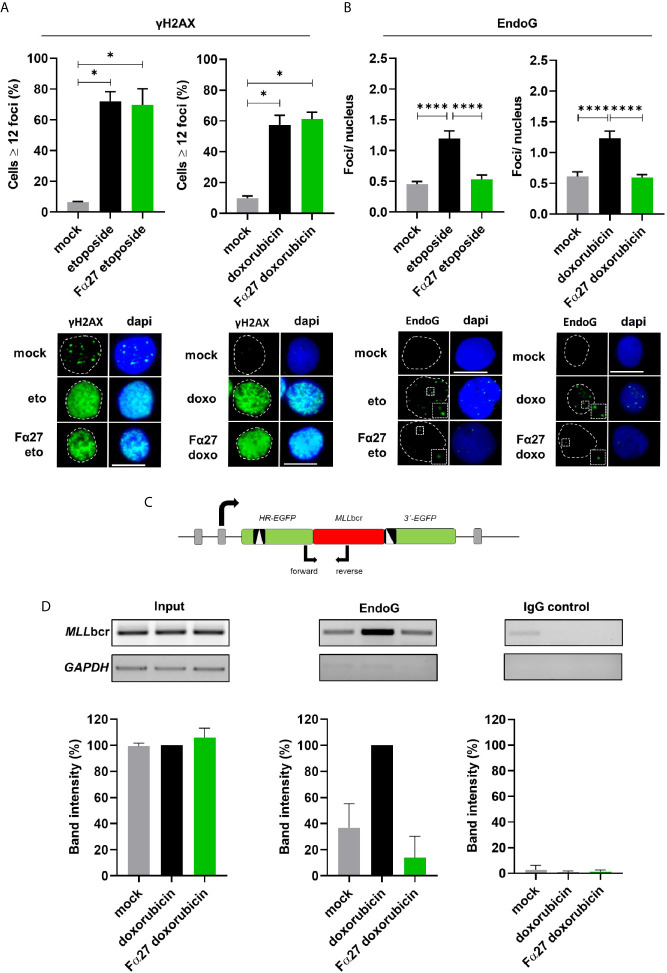
Fα27 influences EndoG accumulation in nuclear foci during treatment and reduces local EndoG binding to the *MLL*bcr. **(A)** No effect of Fα27 on nuclear DNA damage level during chemotherapeutic treatment. WTK1 cells were treated for 4 h with 1 mg/ml Fα27 followed by inclusion of 10 µM etoposide for 4 h or 10 µg/ml Fα27 for 4 h followed by inclusion of 0.5 µM doxorubicin for 4 h. Fixed cells showed an increase of cells with ≥12 γH2AX foci/nucleus after etoposide or doxorubicin treatment but no decrease with Fα27 pre-treatment (n=4 obtained in two independent experiments). **(B)** Decrease of nuclear EndoG signals by Fα27 treatment. WTK1 cells were treated as described in **(A)** and immunofluorescently labeled for EndoG detection. The nuclear EndoG signals increased after etoposide (n=294-463 obtained in two independent experiments) or doxorubicin (n=296-485 obtained in two independent experiments) treatments and Fα27 significantly decreased the nuclear EndoG level. Insets display the highlighted region at two-fold magnification. Scale bars indicate 10 µM. White dashed lines encircle the DAPI-stained nucleus. All values represent mean +SEM (*p < 0.05; ****p < 0.0001). **(C)** Scheme of the *MLL*bcr region flanked by two mutated *EGFP* genes. The scheme visualizes the positions of the primers used for PCR after chromatin immunoprecipitation (ChIP). To analyze the binding of EndoG to the *MLL*bcr a ChIP was performed. Sonified DNA bound by proteins was immunoprecipitated with EndoG antibody or incubated with a control antibody. After DNA isolation a PCR was performed and band intensities evaluated. **(D)** Decreased EndoG binding to the *MLL*bcr after Fα27 treatment. WTK1MLL cells were treated for 4 h with 10 µg/ml Fα27 and additionally with 0.5 µM doxorubicin for 4 h. Two independent experiments for EndoG-ChIP and IgG-ChIP were performed. After PCR amplification of the *MLL*bcr in the recombination reporter, band intensities for input DNA and EndoG-ChIP samples after doxorubicin treatment were set to 100% and relative intensities calculated for the other samples including IgG-ChIP detected on the same membrane and with the same exposure time. EndoG binding to *MLL*bcr was increased after doxorubicin treatment and decreased with Fα27 treatment. Neither the input nor the control showed such a pattern. Control GAPDH PCR was positive for input but negative for ChIP samples. Data are shown as mean +SD. See also **Figure S3**.

Previous works provided evidence for an increase of the mitochondrial nuclease EndoG in the nucleus and a critical involvement of this enzyme in the specific cleavage of the *MLLbcr* during replication stress such as following treatment with the cytostatic drug etoposide (20, 40, 41). Therefore, we examined the impact of Fα27 on these EndoG functions. To firstly verify the accumulation of DNA lesions in the nucleus due to the chemotherapeutic treatments, we immunostained WTK1 cells using antibodies directed against the damage marker γH2AX. Both exposures to etoposide and doxorubicin for 4 h led to sharp rises of nuclear γH2AX signals which were not influenced by Fα27 (**Figure 4A**). When we performed immunofluorescence microscopy with anti-EndoG antibodies, we detected distinct EndoG foci in the nucleus accumulating after the chemotherapeutic treatments (**Figure 4B**). Strikingly, pre-incubation with Fα27 peptide decreased nuclear EndoG signals down to the basal level in untreated cells. To exclude a cell cycle dependent effect of EndoG localization, we performed cell cycle analysis under the conditions of the immunofluorescence microscopic analyses. **Figure S3** reveals that Fα27 pre-incubation did not alter the cell cycle distribution or percentage of apoptotic cells in cells treated with etoposide or doxorubicin.

To analyze the local binding of EndoG on *MLL*bcr sequences we performed chromatin immunoprecipitations (ChIPs) with anti-EndoG antibodies in WTK1MLL cells. PCR amplification of precipitated genomic DNA was specific for the *MLL*bcr region inserted in the chromosomally integrated reporter sequence (**Figure 4C**). WTK1MLL cells showed an increase of EndoG binding to the *MLL*bcr sequence after treatment with doxorubicin (**Figure 4D**), which was decreased to basal levels by Fα27 peptide pre-treatment.

All-in-all, the peptide Fα27 affects the localization of EndoG in the nucleus and at the *MLL*bcr sequence without any influence on the global DNA damage.

### Localization of the Fα27 Peptide

**Figure 5:**
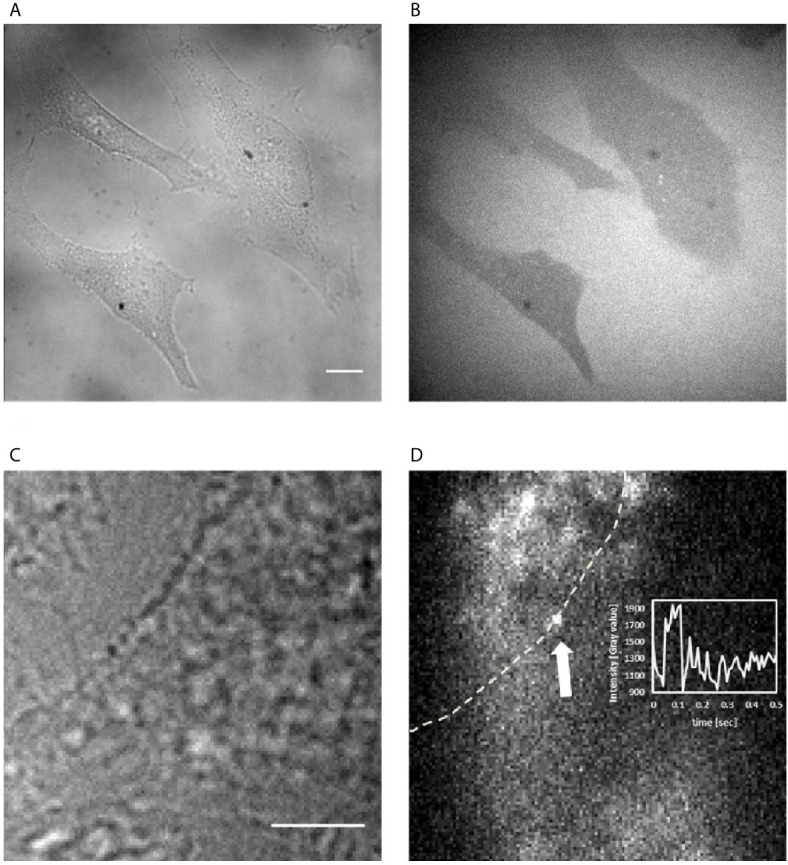
Visualization of Fα27 localization in HeLa cells. **(A)** Wide field image of HeLa cells at 60x magnification to observe cellular outlines and **(B)** image of the same field of view with spinning disc confocal fluorescence microscopy to visualize the localization of TAMRA-Fα27 present at 0.27 µM in the medium. Cells not taking up TAMRA-Fα27 appear dark within the bright background of medium containing TAMRA-Fα27. **(C)** Wide field image of a HeLa cell at 100x magnification to observe the cellular outline and **(D)** image of the same field of view with HILO fluorescence microscopy with single molecule sensitivity to visualize TAMRA-Fα27 present at 2.7 nM in the medium. The white dashed line indicates the cell membrane revealed in **(C)**. The white arrow indicates a single TAMRA-Fα27 molecule visible as bright diffraction-limited spot. Bright intensity in the medium [upper left corner in **(D)**] comes from fast diffusing TAMRA-Fα27 molecules, whose signal is spread out over a larger area during the camera exposure time. Inset: mean intensity *vs* time plot of a small area encircling the spot indicated by the arrow. The signal of TAMRA-Fα27 is visible between ~0.05s and ~0.1s. Scale bar is 10 μm in **(A, B)** and 5 μm in **(C, D)**.

To discriminate between a potential extracellular *vs.* intracellular mode-of-action of Fα27, we visualized its cellular localization upon addition to the culture medium. For these experiments we used adherent HeLa cells optimal for microscopy. We previously demonstrated that HeLa cells generate comparable results to cells from the hematopoietic system in siRNA screenings, functional and biochemical analyses and thus serve as a representative cell model for the processes leading to MLL*bcr* rearrangements (19, 20). We used chemically synthesized Fα27 N-terminally labeled with a TAMRA dye, which is membrane permeable and does not interfere with cellular entry of the peptide. First, spinning disc confocal microscopy revealed dark cells compared to bright, TAMRA dye-positive medium, strongly indicating that Fα27 is unable to cross the cell membrane (**Figures 5A, B**). Second, HILO microscopy, which enables single molecule sensitivity by reducing autofluorescent background of the cell *via* sheet-like illumination (36), allowed us to exclude the presence of potentially active trace amounts of Fα27 in the cellular interior (**Figures 5C, D**). Occasionally, we observed individual labeled peptides associating transiently with the cell membrane.

Overall, these experiments suggested that Fα27 acts by extracellular signaling.

### Fα27 Acts *via* NFκB and Regulates TLR4 Target Genes

**Figure 6:**
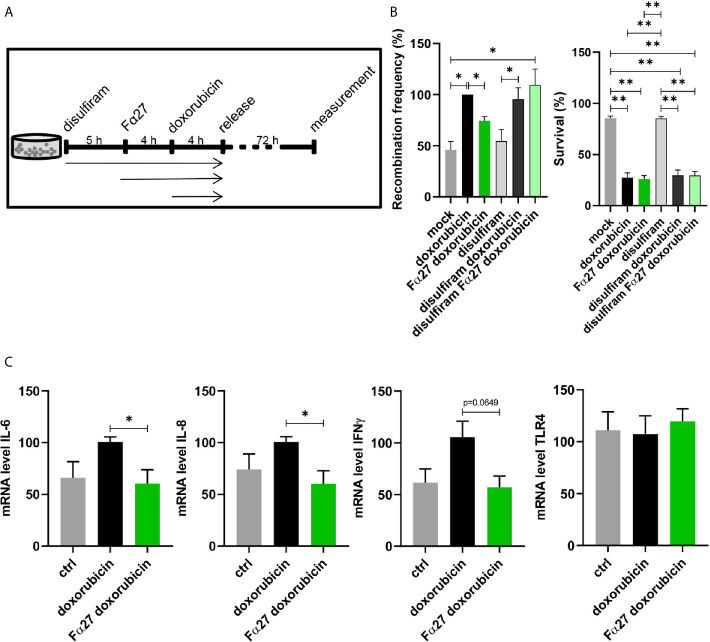
Involvement of NFκB and TLR4 in Fα27 signaling. **(A)** Scheme for doxorubicin treatment protocol following exposure to the NFκB inhibitor disulfiram. WTK1MLL cells were treated for 5 h with 4 µM disulfiram followed by inclusion of 10 µg/ml Fα27 for 4 h and 0.5 µM doxorubicin for another 4 h. Then the cells were released by washing with PBS and seeded in fresh medium. **(B)** Influence of disulfiram under Fα27 and doxorubicin treatment. The recombination frequency measured by FACS showed a decrease of doxorubicin-induced *MLL*bcr rearrangements by Fα27 treatment (six independent experiments). This effect was lost in the presence of disulfiram. Mean recombination frequencies of doxorubicin treated cells were set to 100% each (absolute mean: 1.6x10^-5^). The panel in the right shows that survival was affected by doxorubicin but not by disulfiram. Data are represented as mean +SEM (*p < 0.05; **p < 0.01). **(C)** qRT-PCR of TLR4 downstream genes. The cells were treated with 10 µg/ml Fα27 for 4 h, 0.5 µM doxorubicin included for 4 h, total RNA isolated and mRNA subjected to qRT-PCR. Doxorubicin treatment increased mRNA expression of IL-6, IL-8 and IFNγ, whereas Fα27 reduced these doxorubicin-induced levels (n=5-6 obtained in three independent experiments). TLR4 mRNA expression was not changed. Statistical significances were calculated for doxorubicin-treated cells using Mann-Whitney-U test (*p < 0.05) and values are represented as mean +SEM. See also **Figures S4** and **S5**.

Landers et al. (42) reported that fibrinogen, particularly the fibrinogen α chain, is selectively cleaved by proteinases and resulting cleavage products can bind TLR4 and regulate downstream transcription factors like nuclear factor kappa-light-chain-enhancer of activated B-cells (NFκB). Though fibrinogen α fragments identified by Landers and colleagues were not identical with Fα27, we were inspired by these findings and therefore analyzed involvement of NFκB signaling in the pathway regulated by Fα27 by use of the NFκB inhibitor disulfiram (43). Disulfiram was added to the medium of WTK1MLL cell cultures as the first pre-treatment, i.e. 5 h before sequential Fα27 and Fα27 plus doxorubicin pre-incubations followed by release into fresh medium (**Figure 6A**). Recombination measurements showed similar basal frequencies and increases in doxorubicin-exposed WTK1MLL cells with and without disulfiram (**Figure 6B**). Fα27 exposure reduced the recombination frequency in the absence but no longer in the presence of disulfiram (**Figure 6B**). The Fα27 effect on recombination was also abrogated in etoposide-exposed cells, even though combined disulfiram and etoposide treatment stimulated etoposide-induced *MLL*bcr rearrangements and reduced survival even further compared with single etoposide treatment (**Figure S4**). Of note, accumulation of DNA damage and/or compensatory use of alternative DSB repair pathways may explain why *MLL*bcr rearrangements continued after inhibition of NFκB, which promotes non-homologous end joining (NHEJ) and HR (18, 31).

Given that ROS trigger NFκB signaling (44, 45) and that ROS production is one of the major toxic effects of chemotherapeutic treatments, doxorubicin in particular (46), we investigated a potential role of ROS in the Fα27 effect. When exposing the cells to N-acetylcysteine (NAC), a superoxide scavenger (47) that decreases ROS production, the Fα27-dependent decrease in the recombination frequency of doxorubicin-treated WTK1MLL cells was no longer detectable (**Figure S5**).

To test the hypothesis that proinflammatory signaling by TLR4 and NFκB is influenced by Fα27, we checked the expression of characteristic cytokines downstream of this pathway. The mRNA levels of interleukin (IL)-6, IL-8 and interferon (IFN)γ all increased after doxorubicin treatment and decreased following Fα27 pre-treatment (**Figure 6C**). The TLR4 mRNA expression level was not changed under these conditions.

Altogether, both recombination measurements when inhibiting NFκB and analysis of TLR4 activated genes support our hypothesis that TLR4 signaling is blocked by Fα27.

### Fα27 Antagonizes LPS-Mediated TLR4 Activation

**Figure 7:**
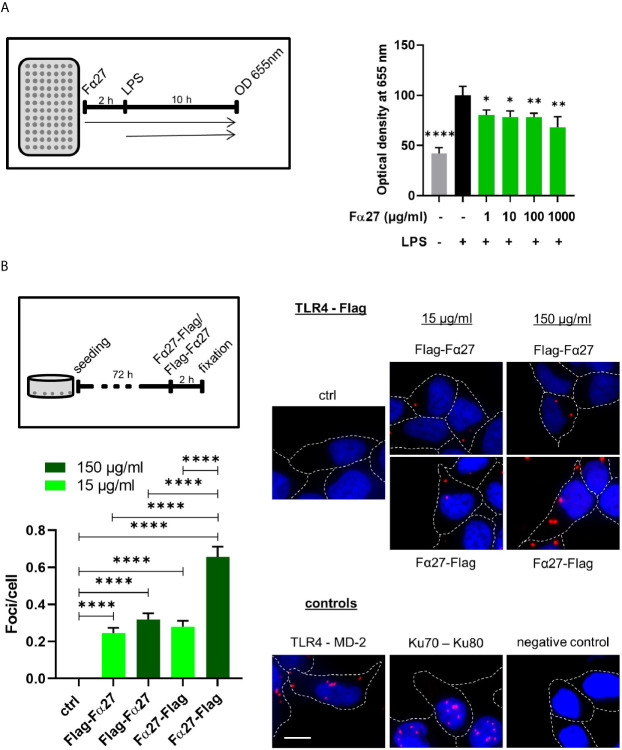
Fα27 binds TLR4 and reduces LPS stimulation. **(A)** Fα27 inhibits TLR4 activation by LPS. Scheme of experimental analysis of TLR4 signaling and results obtained in HEK-Blue hTLR4 cells pre-treated for 2 h with different concentrations of Fα27 followed by additional treatment with 10 ng/ml LPS for 10 h. TLR4 stimulation led to a release of secreted embryonic alkaline phosphatase (SEAP) detected at OD 655 nm. LPS treatment increased SEAP release which was reduced by Fα27 pre-treatment. Mean OD values of LPS-treated controls without peptide were set to 100% in each experiment. Data (n=12 obtained in two experiments) are shown as mean +SEM and statistics were calculated relative to LPS stimulation without peptide (*p < 0.05; **p < 0.01; ****p < 0.0001). **(B)** TLR4 and Fα27 interaction. HEK-Blue hTLR4 cells were seeded 72 h before the experiment and treated for 2 h with N-terminally tagged Flag-Fα27, C-terminally tagged Fα27-Flag or water (ctrl). PLA analysis showed an interaction between both tagged Fα27 peptides and the TLR4. Two positive controls are shown: PLA for TLR4 and its binding partner MD-2 on the cellular surface and for Ku70-Ku80 complexes in the nucleus. The negative control was done with PLA substrates but without antibodies. Margins of cells are marked by white stippled lines and the scale bar indicates 10 µM (n=187-425 from two independent experiments each; mean +SEM; ****p < 0.0001). See also **Figure S6**.

To examine whether Fα27 directly targets TLR4, we studied the impact of Fα27 on TLR4 signaling after extracellular stimulation by the well-established TLR4 agonist lipopolysaccharide (LPS). To this end we engaged HEK-Blue™ hTLR4 reporter cells enabling TLR4 activity measurements *via* a color change due to inducible release of SEAP (secreted embryonic alkaline phosphatase). Our results showed that pre-treatment with Fα27 decreased LPS stimulation of TLR4 significantly at different peptide concentrations by 19% to 32% (**Figure 7A**). For comparison, when HEK-Blue™ hTLR4 cells were pre-treated with Scramble 2, composed of the Fα27 amino acids in a random order, LPS stimulation was not significantly compromised (**Figure S6A**).

To examine a potential influence of Fα27 on TLR4 signaling during chemotherapeutic treatment, we treated HEK-Blue™ hTLR4 cells with doxorubicin after pre-treatment with active or control peptides (**Figure S6B**). Doxorubicin treatment on its own did not affect the release of the reporter SEAP. In the presence of peptides, we observed a statistically significant decrease of TLR4 signaling by 21% when comparing Fα27 versus Scramble 2 pre-treated cells.

In summary, Fα27 antagonizes TLR4 activation by LPS in a sequence-specific fashion, suggesting a direct interaction of Fα27 with TLR4.

### Detection of a Close Proximity Between TLR4 and Fα27 *In Situ*

To test the hypothesis of an association of Fα27 with TLR4, we examined whether they can be found in close proximity to each other. Therefore, we labeled Fα27 either N- or C-terminally with a Flag-Tag, which was separated from the peptide by a GSSGSS-linker each. Proximity ligation assay (PLA) studies with HEK-Blue™ hTLR4 cells revealed sites of proximity between TLR4 and both Fα27 versions spread over the cellular area, similarly as seen for TLR4 and its co-receptor myeloid differentiation 2 (MD-2) (**Figure 7B**). To strengthen this result, the corresponding experiment was also performed with WTK1 cells showing comparable results (**Figure S7**). For comparison, PLA signals for the DNA repair proteins Ku70 and Ku80 were limited to the DAPI-stained nucleus. Raising the concentration of tagged Fα27 increased the average numbers of foci per cell, reaching statistical significance after incubation with C-terminally tagged peptide Fα27-Flag.

### Human Hematopoietic Stem and Progenitor Cells Show Increased Breakage of the *MLL*bcr Under Doxorubicin Treatment Which Is Prevented by Fα27

**Figure 8:**
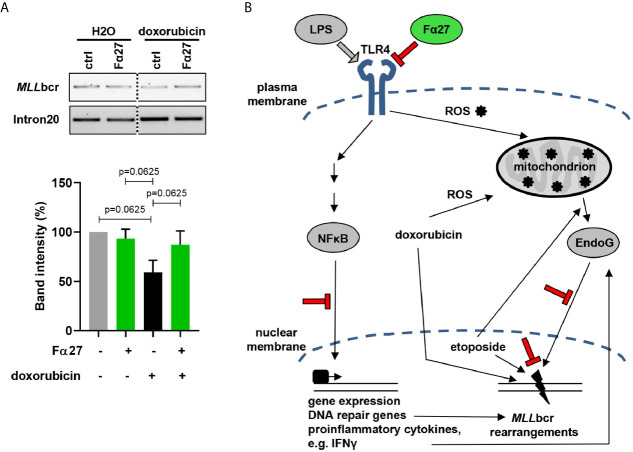
Fα27 prevents *MLL*bcr breakage in HSPCs. **(A)** *MLL*bcr breakage analysis after doxorubicin and Fα27 treatment of HSPCs. HSPCs were treated 4 h with 10 µg/ml Fα27 followed by 4 h of treatment with Fα27 plus 0.072 µM doxorubicin. After genomic DNA isolation breakage was assessed by PCR targeting the endogenous *MLL*bcr locus. Doxorubicin treatment increased breakage as indicated by a 40% decrease of the band intensity, which was reduced in Fα27 pre-treated cells by only 13%. Data from five independent experiments are shown as mean +SEM (Friedman-Test indicating p=0.0087, followed by Wilcoxon test). The framed image shows *MLL*bcr PCR amplifications from the same gel exposed with the same intensity and cropped at the dotted line. *MLL* intron20 was amplified as control. **(B)** Model of *MLL*bcr protection by Fα27. LPS activation of TLR4 leads to NFκB-mediated gene expression of DNA repair genes and proinflammatory cytokines like type I interferons (51). The interferon IFNγ can increase EndoG release from the mitochondria (52). Etoposide, a topoisomerase II inhibitor, induces DNA damage and EndoG release from the mitochondria (53). Doxorubicin, a drug with a broad spectrum of cell damage, intercalates into the DNA, which promotes translocation of EndoG from mitochondria to the nucleus. Nuclear EndoG cleaves the *MLL*bcr predicted to form pronounced secondary structures. Stimulation of TLR4, such as by LPS, was as well reported to increase ROS production in mitochondria (54), again inducing DNA damage. We show that Fα27 blocks TLR4 activation and downstream NFκB signaling. Furthermore, EndoG accumulation in the nucleus is reduced and the *MLL* gene protected from breakage. Findings made in this work are indicated in red color.

Hematopoietic stem and progenitor cells harbor the cells-of-origin of leukemia (48, 49) and express TLRs and their co-receptors (50). To examine the impact of Fα27 on *MLL*bcr stability in these cells, we treated primary human cord blood-derived HSPCs with doxorubicin at the IC80 dose of 0.072 µM. Treatment of HSPCs with doxorubicin caused breakage of approximately half of the *MLL*bcr sites, i.e. caused by 40% reduced band intensities after genomic PCR amplification of the *MLL*bcr as compared to the distant intron 20 control site within the *MLL* gene (**Figure 8A**). Strikingly, pre-treatment with Fα27 reduced breakage as reflected by a 13% reduction of the PCR band intensities only. From this we concluded that Fα27 not only prevents *MLL*bcr rearrangements in immortalized cells derived from the human hematopoietic system but also protects *MLL*bcr from breakage in primary human HSPCs.

## Discussion

In this study, we screened a hemofiltrate-derived library for peptides decreasing leukemic *MLL*bcr rearrangements and identified a peptide with 27 amino acids derived from the C-terminus of the fibrinogen α chain (Fα27). Fibrinogen, composed of the α, β and γ chains, is up-regulated and cleaved during inflammation as well as after vascular injury to form fibrin, a component of blood clots (55, 56). Further reports described increased expression of fibrinogen in a large variety of tumors (57–59) and correlation of high fibrinogen plasma levels with cancer risk, worse disease-free and poor overall survival (59–61). Peptides derived from the C-terminus of fibrinogen α (62–64), including a peptide identical to Fα27 (65), were also identified during clinical association studies in search of serum-derived tumor biomarkers. Notably, one study focusing on acute myeloid leukemia (AML) showed that a 38 amino acid long C-terminal fragment of fibrinogen α encompassing Fα27 was found to be reduced in sera from newly diagnosed, refractory and relapsed cases and associated with improved overall survival of patients, which was the opposite of what was observed for the fibrinogen α level (62). In our work, we discovered Fα27 to show sequence specific bioactivity with IC50 values in the lower micromolar, i.e. the physiological range of fibrinogen (66). Importantly, we provide evidence that Fα27 protects against *MLL*bcr breakage and rearrangements triggering AML, but not against global DNA damage or cell death induced by cytostatic treatments and therefore holds promise to ameliorate side effects without compromising the therapeutic achievement.

After identification of the bioactive peptide in the circulating blood peptidome we confirmed functional equivalence of its synthetic counterpart and analyzed its mode-of-action. Wide field image microscopy of TAMRA-labeled Fα27 showed that the peptide was not entering the cell. Therefore, we considered that Fα27 acts *via* a membrane receptor. Smiley and colleagues (67) reported that fibrinogen activates TLR4 on macrophages. More recent work by Landers and colleagues (42) showed that fibrinogen α undergoes proteolytic processing releasing C-terminal regions during innate immune responses and generating cleavage products that can directly bind TLR4. In the light of previous observations suggesting that full-length fibrinogen and C-terminal peptides may exert opposing effects during carcinogenesis (62), it is tempting to speculate that these peptides interfere with fibrinogen functions. The protruding and flexible fibrinogen α C-terminal regions can self-interact both within and between fibrinogen protofibrils and these interactions play a role in polymer formation (68). Though the precise mechanism of this cross-talk will have to be elucidated in future studies, C-terminal peptides like Fα27 could aggregate with fibrinogen and, thus, affect the interaction of fibrinogen with TLR4 and co-receptors (42, 69).

A variety of molecules activate TLRs on the cell surface (69). TLR4 is best known to be targeted by LPS from bacteria, which leads to cytokine production (70). We therefore analyzed the effect of Fα27 on LPS-activated TLR4 signaling using a reporter cell line and indeed noticed an inhibitory effect of the peptide in the concentration range exerting *MLL*bcr protection. Importantly, scrambled peptide was ineffective excluding that simple physicochemical characteristics of Fα27 underly the inhibitory interaction such as known from positively charged antimicrobial peptides (71). In support of a physical interaction between Fα27 and TLR4, we detected PLA signals by immunofluorescence microscopy. This was found to be true particularly for C-terminally labeled peptide, i.e. labeled at the terminus where in the full-length fibrinogen α molecule only 15 further amino acids form part of the flexible C-terminal tail. With increasing peptide concentration, PLA foci for TLR4 and Fα27 reached half of the numbers per cell counted for TLR4 and its co-receptor MD-2.

These results together with our data obtained on (i) the nuclear localization and *MLL*bcr interactions of EndoG, (ii) *MLL*bcr rearrangements after NFκB inhibition or ROS scavenging as well as (iii) cytokine expression led to our model proposed in **Figure 8B**. Accordingly, Fα27 blocks TLR4 activation by its ligands such as LPS, which involves association of the peptide with TLR4, its co-receptors or its ligands. In this way Fα27 affects intracellular signaling mechanisms downstream of TLR4, all merging at *MLL*bcr breakage and rearrangements:

First, dampening the NFκB pathway compromises DSB repair *via* NHEJ and HR, two pathways contributing to chromosomal rearrangements (18, 19, 31). In our study, use of the pharmacological inhibitor disulfiram showed that Fα27 targets the NFκB pathway when counteracting etoposide- and doxorubicin-induced *MLL*bcr rearrangements. Analysis of I-*Sce*I-induced repair revealed that Fα27 affects the DSB repair process itself, yet moderately, and therefore can only partially explain Fα27 mode-of-action. Yet, among the different DSB repair genes NFκB transcriptionally up-regulates also ATM, which not only regulates DSB repair but also promotes RNF20-RNF40-mediated H2B ubiquitination and chromatin remodeling as a prerequisite for EndoG loading onto the *MLL*bcr (20). Thus, the *MLL*bcr protective effect of Fα27 may as well influences NFκB´s involvement in other cellular activities ranging from chromatin remodeling to cytokine production.

Second, inflammatory signaling is known to generate ROS (72, 73), inducing transport of EndoG from mitochondria to the nucleus, where EndoG binds and cleaves the *MLL*bcr (19). Along this line, the proinflammatory cytokine IFNγ was reported to promote EndoG release from mitochondria (52) following TLR4 activation. Here, we observed that Fα27 inhibits LPS-activated TLR4 signaling and expression of the proinflammatory cytokines IL-6, IL-8 and IFNγ. Therefore, blocking inflammatory signaling and ROS production represents another mechanism contributing to *MLL*bcr protection by Fα27.

Moreover, chemotherapeutic treatment itself causes the accumulation of ROS. Particularly doxorubicin boosts ROS formation in mitochondria (21, 39), but also etoposide was shown to shuttle EndoG into the nucleus (53). In response to chemotherapeutic treatment, we saw focal accumulation of EndoG in the nucleus and *MLL*bcr binding, which was counteracted by Fα27. In the presence of the ROS scavenger NAC, treatment-induced rearrangements at the *MLL*bcr were no longer suppressed by Fα27. The peptide may therefore break a loop amplifying ROS production by the chemotherapeutic treatment and TLR4 signaling.

What is the relative importance of these mechanisms underlying the protective mode-of-action of Fα27? Clues may come from the activities of cells treated with etoposide *versus* doxorubicin. While both drugs are topoisomerase II inhibitors, etoposide forms topoisomerase poisoning complexes, blocking DNA resealing during the enzymatic reaction, whereas doxorubicin intercalates into the double helix compromising topoisomerase functions, RNA- and DNA synthesis as well as generating free oxygen radicals (38, 39). Therefore, etoposide primarily induces DSBs, while doxorubicin additionally causes formation of oxidative DNA lesions, DNA adducts and RNA-DNA hybrids, representing obstacles for nascent DNA synthesis (74). Interestingly, when we studied *MLL*bcr rearrangements following a short (4 h) treatment period and a long (72 h) recovery period versus uninterrupted treatment, a recovery period was necessary to unveil the protective effect of Fα27 during doxorubicin but not etoposide treatment. These results suggested that Fα27 antagonizes formation of doxorubicin-induced DNA damage at the *MLL*bcr rather than its repair, while in case of etoposide the impact of Fα27 on the repair of drug-induced DSBs at the *MLL*bcr is as well significant. Yet, the effect of the peptide on DSB repair following targeted cleavage of the chromosomal reporter constructs was moderate, which may explain why reduction of *MLL*bcr rearrangements required a higher peptide concentration after etoposide than after doxorubicin exposures. Accordingly, we propose that Fα27 is most potent in *MLL*bcr protection, when the *MLL*bcr is challenged by synergistically destabilizing mechanisms such as during doxorubicin treatment.

Knowing that Fα27´s effect on the *MLL*bcr involves oxidative stress and proinflammatory signaling *via* TLR4 and NFκB, how then are the two pathways connected? In this context reports showing up-regulation of the expression level of TLR4 by doxorubicin are of interest (75, 76). This upregulated level of TLR4, though not reaching statistical significance at early time points in mice or when comparing patient cohorts, has been discussed to contribute to toxic side effects in chemotherapy like cardiomyopathy (76). Moreover, published work demonstrated that doxorubicin-mediated DNA damage results in the production of single-stranded DNA that directly activates the DNA-sensing cGAS-STING pathway of IFN induction (77). Indeed, TLRs are not only activated by so-called pathogen-associated molecular patterns (PAMPs) but also by damage-associated molecular patterns (DAMPs). While PAMPs like bacterial LPS and virus-derived nucleic acids stem from exogenous sources, namely microbiological infections, DAMPs are intracellular molecules such as host DNA or fibrinogen, which are released by activated or dying cells (72, 78). Though conflicting evidence regarding the precise binding sites of PAMPs and DAMPs exist, there is a possibility that LPS and fibrinogen compete for binding to the same binding site on TLR4. Doxorubicin treatment therefore triggers TLR4 signaling *via* DAMPs from dying cells as well as *via* upregulation of the receptor itself. Inflammatory TLR4 signaling will further increase tissue damage and therefore DAMPs. In this way, an amplification loop of signals destabilizing the *MLL*bcr through IFN and extra ROS generated during TLR4 signaling is created, which ultimately shuttles EndoG to the nucleus where it specifically cleaves hard-to-replicate sites such as the *MLL*bcr in surviving cells (20). Importantly, HSPCs are highly sensitive to products which activate TLRs and respond to type I and II interferons, like IFNγ. Consistently, we observed that Fα27 pre-treatment ameliorates *MLL*bcr breakage in HSPCs during doxorubicin chemotherapy. After bone marrow transplantation 13% of the patients were reported to develop a secondary malignancy in the first 15 years after treatment (79). Breaking the cascade of proinflammatory signaling events directly at the receptor *via* an antagonistic fibrinogen fragment therefore has potential to specifically reduce the side effect of leukemic rearrangements in the cells-of-origin of leukemia.

Our work provides proof-of-concept that genome destabilizing events causing secondary leukemia can be prevented by a peptide blocking TLR4 activation. Since Fα27 is an endogenous peptide, it is conceivable that an autoregulatory feedback loop involving Fα27 and related fibrinogen α-derived peptides exists. Thus, at sites of tissue injury and inflammation upregulation of DAMPs like fibrinogen will activate TLR4 to initiate inflammation; proteolytic cleavage products of fibrinogen may thereafter antagonize TLR4 activation and terminate inflammation. Loss of such control mechanisms will deregulate TLR signaling and contribute to the persistence of inflammatory responses underlying chronic diseases (80, 81). Persistent DAMP accumulation and inflammation are associated with cancer (72). TLR4 signaling has been shown to enhance the proliferation and migration of malignant tumor cells in patients (82, 83), and overexpression in tumors was correlated with metastasis and chemoresistance (84). Our work has provided evidence for even another detrimental outcome of TLR4 signaling in cancer patients undergoing chemotherapy, i.e. leukemia-inducing chromosomal damage in healthy HSPCs without killing the cells. Given that TLR4 is unique among TLRs in terms of its MD-2 co-receptor requirement (85), it has increasingly gained interest as a target of therapeutic strategies engaging small molecules (72, 86, 87). Since Fα27 is an endogenous inhibitor of TLR4 signaling that may have undergone natural selection processes, Fα27 could be of interest as a lead compound that may offer a means to selectively inhibit the DAMP-induced TLR4 activation loop causing secondary leukemia in cancer patients undergoing chemotherapy.

## Data Availability Statement

The original contributions presented in the study are included in the article/**Supplementary Material**. Further inquiries can be directed to the corresponding author.

## Ethics Statement

The studies involving human participants were reviewed and approved by University of Ulm. Written informed consent to participate in this study was provided by the participants’ legal guardian/next of kin.

## Author Contributions

LW conceived the study. RW and BG performed the screen. RW and LM established assay conditions and performed initial experiments. LW and JE designed the experiments. JE performed the experiments and processed the data with help of RW. JG and AP designed, performed and interpreted spinning disc confocal and Hilo microscopy experiments. LS, JM, and WF were in charge of the peptide library and peptide identification. LW and JE interpreted the data and designed the manuscript. JE generated the figures and wrote the initial draft of the manuscript, which was revised by LW. All authors contributed to the article and approved the submitted version.

## Funding

LS, JM, JG, and LW were supported by grants from the German Research Foundation (DFG) within the CRC 1279. LW was further funded by the DFG, in the Research Training Group 1789, and by the German Cancer Aid, Priority Program ‘Translational Oncology: DETECT CTC: Detection and characterization of circulating tumor cells and tumor markers in advanced breast cancer in the context of tumor heterogeneity within the DETECT study program,’ 70112504. LM received a Dr.med. scholarship for Experimental Medicine by Ulm University.

## Conflict of Interest

The authors declare that the research was conducted in the absence of any commercial or financial relationships that could be construed as a potential conflict of interest.
