# Supplementary material for: A Fibrinogen Alpha Fragment Mitigates Chemotherapy-Induced MLL Rearrangements
Source: Front Oncol. 2021 Jun 18;11:689063. doi: 10.3389/fonc.2021.689063 (PMC8249925; doi:10.3389/fonc.2021.689063)
Supplement: Supplementary file 1 [file DataSheet_1.pdf]

2. Supplementary Data

Material and methods

Cell cycle analysis

Cells were treated with Fα27 for 4 h followed by Fα27 plus etoposide or doxorubicin incubation for another 4 h. For fixation cells were washed with PBS (Gibco/Thermo Fisher Scientific, Waltham, Massachusetts, USA) and resuspended in 500 µl PBS. 9 ml ice-cold fixative (80% ethanol/100% acetone from Sigma-Aldrich/Merck, St. Louis, Missouri, USA) 1:1 in water was slowly dropped into the cell suspension during gentle vortexing. After >15 min incubation on ice, the samples were stored at -20°C. Before propidium iodide (PI) staining cells were washed twice, once with PBS/fixative 1:1 and once with PBS. Finally, the cells were resuspended in 200 µl PI solution in PBS (50 µg/ml from Sigma-Aldrich/Merck, St. Louis, Missouri, USA) including 125 ng/ml RNaseA (OriGene, Rockville, Maryland, USA). After a 30 min incubation period at 37°C the cellular DNA content was measured flow cytometrically using FACSCalibur™ (BD Biosciences, San Jose, California, USA).

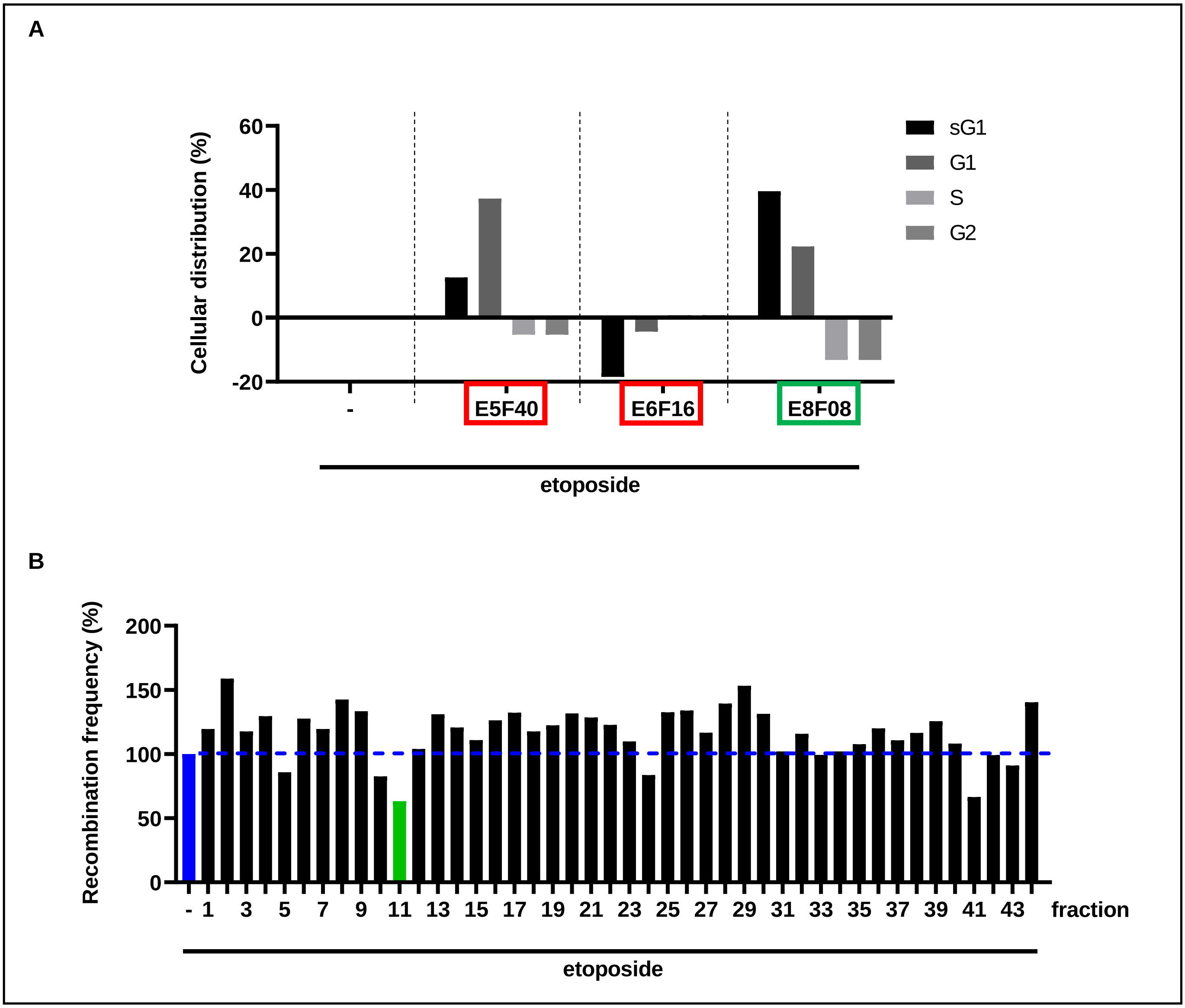

Supplement S1. Changes in DNA content by active fractions and subfractionation of active fraction E8F08.

(A) Cellular distribution in the cell cycle following exposure to fractions inducing significant changes in recombination frequencies. WTK1MLL cells were cultured in 1 ml medium with 10 µl of primary chromatographic fractions (0.2 l hemofiltrate equivalent) for 4 h followed by a 10 µM etoposide treatment for 72 h, fixation and PI staining. The cellular DNA content, measured by FACS, showed moderate changes in apoptosis and the cell cycle distribution. Only fraction E8F08 caused a decrease in recombination (green frames). **(B)** Recombination measurements after subfractionation of E8F08 by reverse phase HPLC. WTK1MLL cells were cultured in 1 ml medium with 10 µl of secondary chromatographic fractions (0.25 l hemofiltrate equivalent) for 4 h and then 72 h additionally including 10 µM etoposide. EGFP positive cells among living cells were measured by FACS. Fraction 11 caused the most pronounced decrease (40%), whereby a neighboring fraction was also active. Mass spectrometry analysis of fraction 11 identified a C-terminal part of Fibrinogen α shown in Figure 1C.

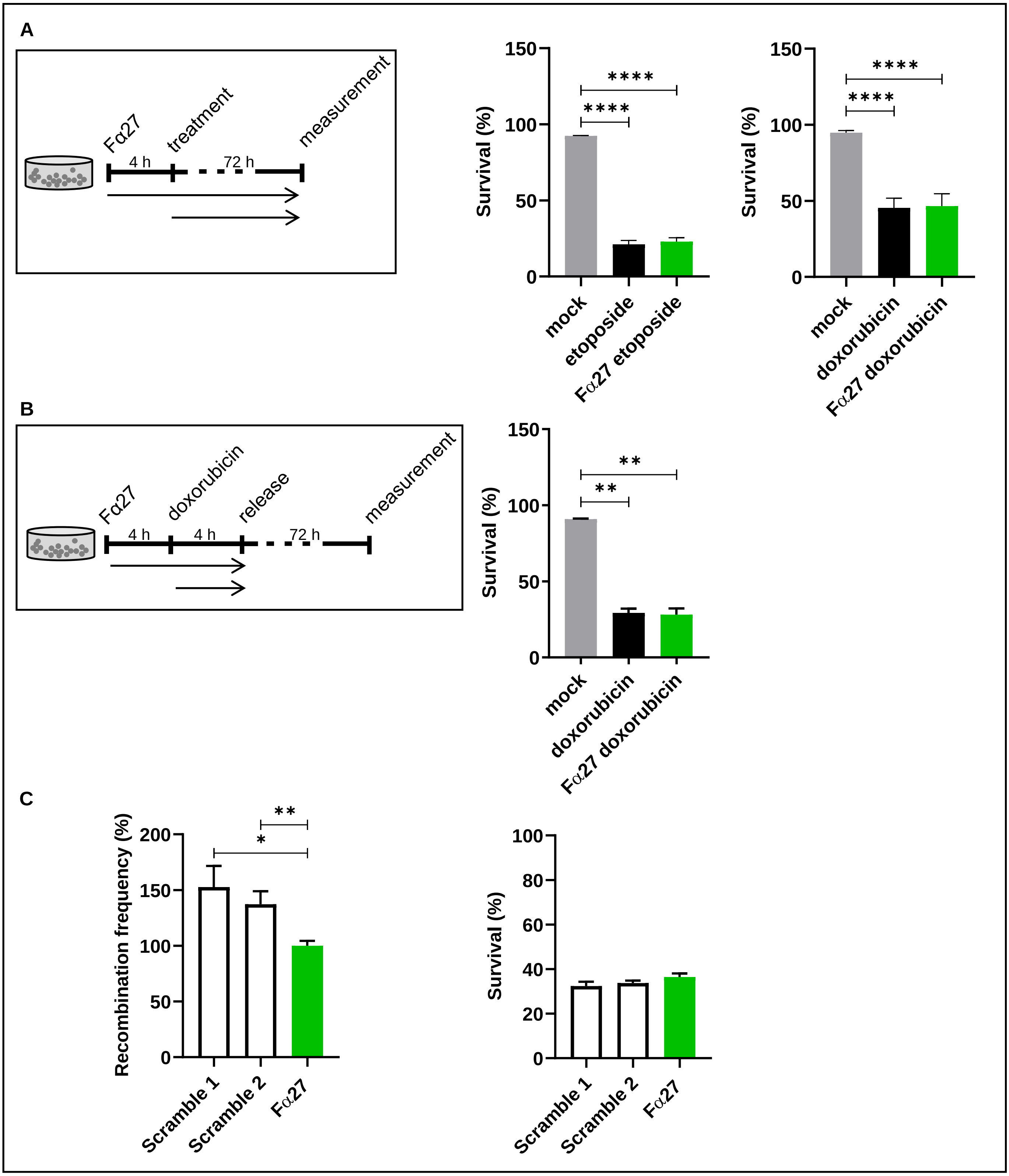

Supplement S2. Fα27 has no influence on survival and shows a sequence specific effect.

Supplement S2. Fα27 has no influence on survival and exerts a sequence-specific effect (Supplement for Figure 2).

(A) Treatment scheme and survival results of WTK1MLL cells following continuous exposure to peptide and chemotherapeutic drug. Survival as determined by FACS analysis (living cells identified in the forward scatter, FSC, versus sideward scatter, SSC, gate) was significantly decreased both after 10 µM etoposide and 0.5 µM doxorubicin treatment (n=12 obtained in four independent experiments). No additional effect of Fα27 (1 mg/ml for etoposide and 10 µg/ml for doxorubicin) was seen. **(B)** Treatment scheme and survival results of WTK1MLL cells following release from exposure to peptide and chemotherapeutic drug. Survival was significantly reduced after 0.5 µM doxorubicin treatment and remained unaffected by Fα27 pre-treatment (n=6 obtained in two independent experiments). **(C)** Comparative analysis of Fα27 with scrambled peptides of 27 amino acids. Peptide Scramble 1 was designed randomly reflecting the amino acid composition in human proteins. Scramble 2 was composed of Fα27 amino acids in a random order. For analysis of their effect on *MLL*bcr rearrangements WTK1MLL cells were treated as schematically outlined in **(B)**, left panel. Thus, the cells were pretreated with 10 µg/ml peptide followed by combined treatment with 0.5 µM doxorubicin for 4 h and a release for 72 h. As compared with Scramble 1 and Scramble 2 treated cells Fα27 showed a decrease in recombination by 35% and 27%, respectively (n=9 obtained in three independent experiments). Data represent mean +SEM (* $p<0.05$; ** $p<0.01$, **** $p<0.0001$).

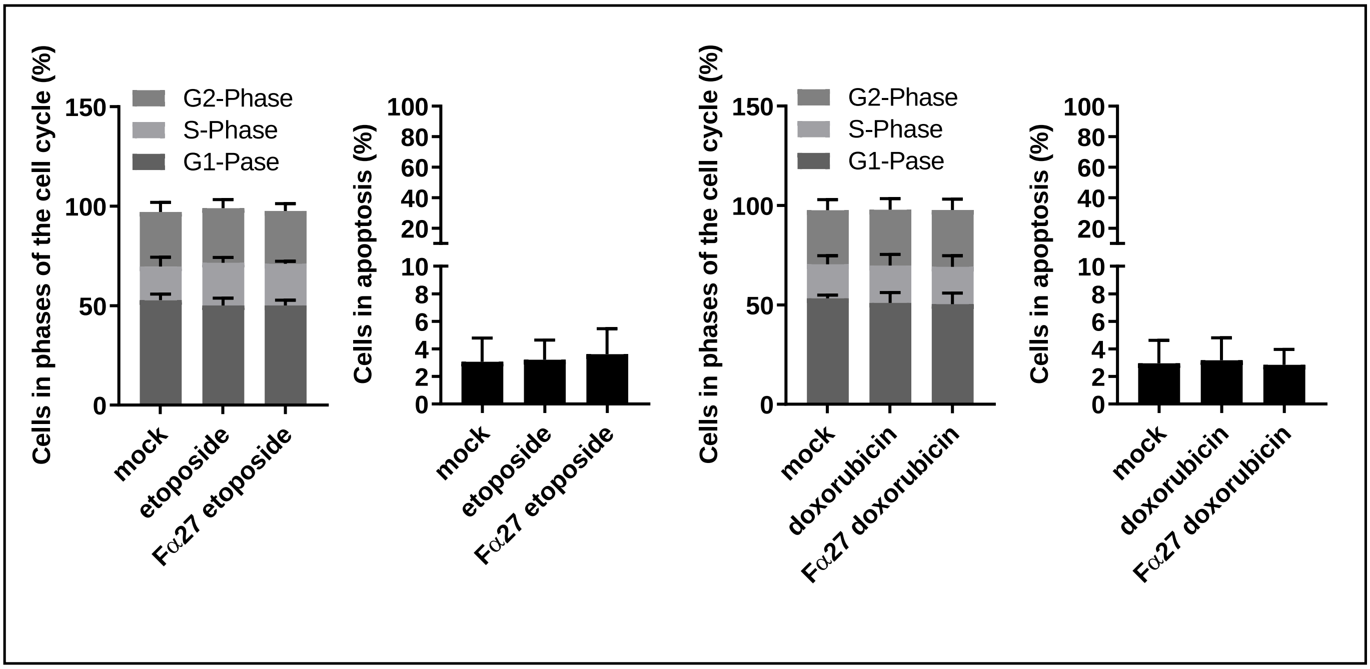

Supplement S3. Fα27 has no influence on the cell cycle (Supplement for Figure 4).
Cell cycle distribution of WTK1MLL cells. Treatment of WTK1MLL cells lasted 4 h for 1 mg/ml Fα27 and 4 h for Fα27 plus 10 µM etoposide exposure or 4 h for 10 µg/ml Fα27 and 4 h for Fα27 plus 0.5 µM doxorubicin exposure. Neither etoposide nor doxorubicin pretreatment with Fα27 changed the cell cycle distribution after 4 h. All data are shown as average of three experiments (mean +SD).

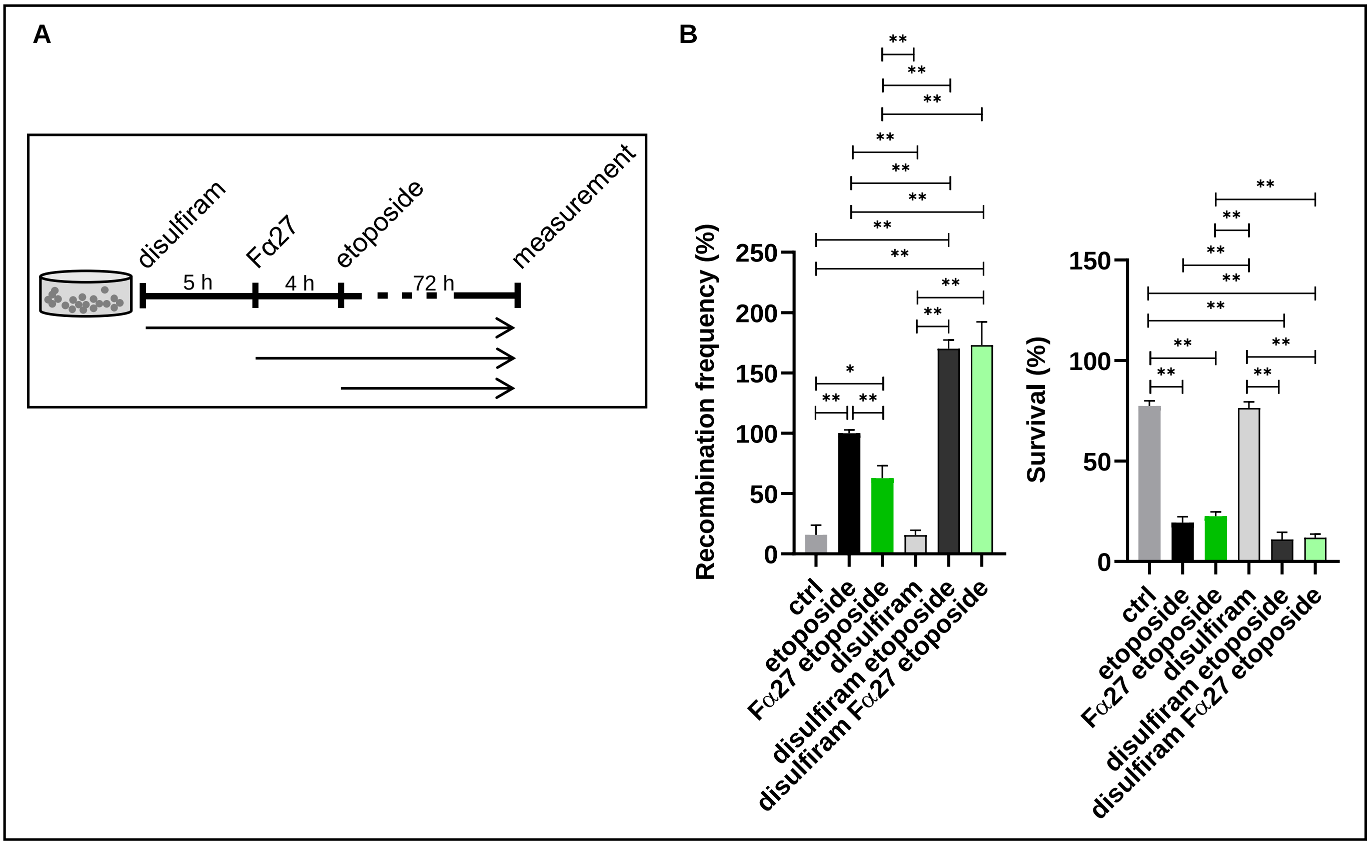

Supplement S4. Involvement of NFκB in Fα27 signaling during etoposide treatment (Supplement for Figure 6B).

(A) Scheme for etoposide treatment protocol following exposure to the NFκB inhibitor disulfiram. WTK1MLL cells were treated for 5 h with 4 µM disulfiram followed by inclusion of 1 mg/ml Fα27 for 4 h and subsequently of 10 µM etoposide for 72 h. **(B)** Influence of disulfiram under Fα27 and etoposide treatment. Fα27 reduced etoposide-induced recombination after treatment as demonstrated also in Figure 2 **(A)**. Disulfiram increased the recombination frequency after etoposide treatment and prevented the mitigating effect by Fα27. Mean recombination frequencies of etoposide treated cells were set to 100% each (absolute mean: $6.2x10^{-5}$). The panel on the right shows that the etoposide-induced decrease of survival was further decreased by disulfiram but not Fα27 exposure. Data (n=7 obtained in three independent experiments) are presented as mean +SEM (*p<0.05; **p<0.01).

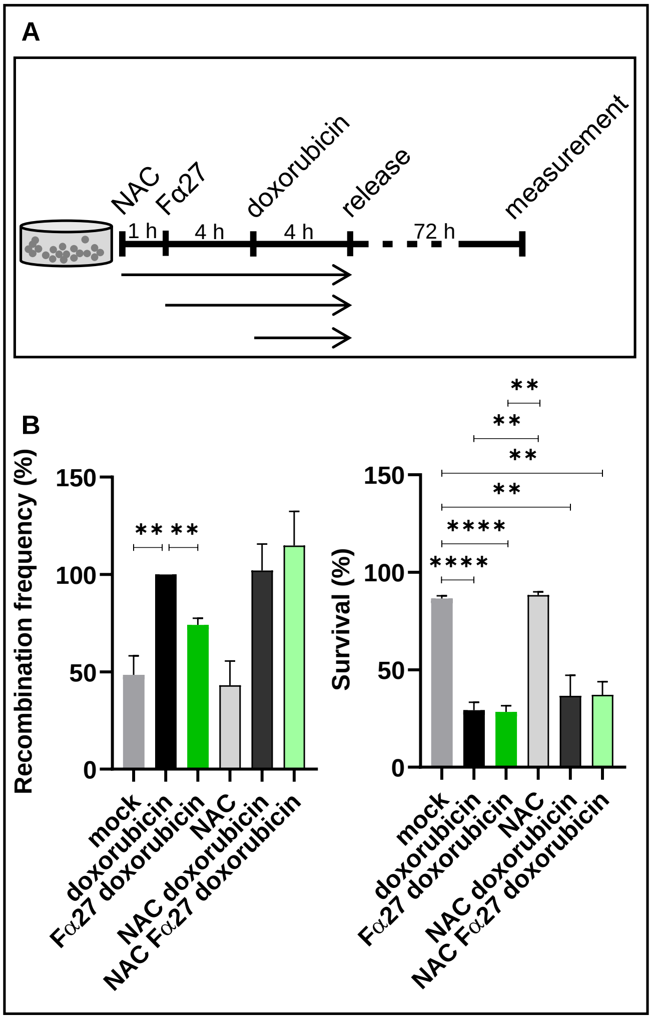

Supplement S5. Involvement of ROS in Fα27 signaling in WTK1MLL cells.

(A) Scheme for N-acetylcysteine (NAC) treatment with release. WTK1MLL cells were treated for one hour with 5 mM NAC, a reactive oxygen species (ROS) scavenger. After the inhibitor treatment the cells were additionally treated for 4 h with 10 µg/ml Fα27 and 4 h additionally with 0.5 µM doxorubicin. Then, the cells were released by washing with PBS and seeded in fresh medium for the recombination measurement. **(B)** Influence of ROS on Fα27 signaling. Cells which were pretreated with NAC showed no longer an effect of Fα27 on the recombination frequency (n=3-9 independent experiments). The control is corresponding to an average recombination frequency of 1.4×10^{-5}. Survival was not affected by NAC. Data are represented as mean +SEM (**$p<0.01$; ****$p<0.0001$).

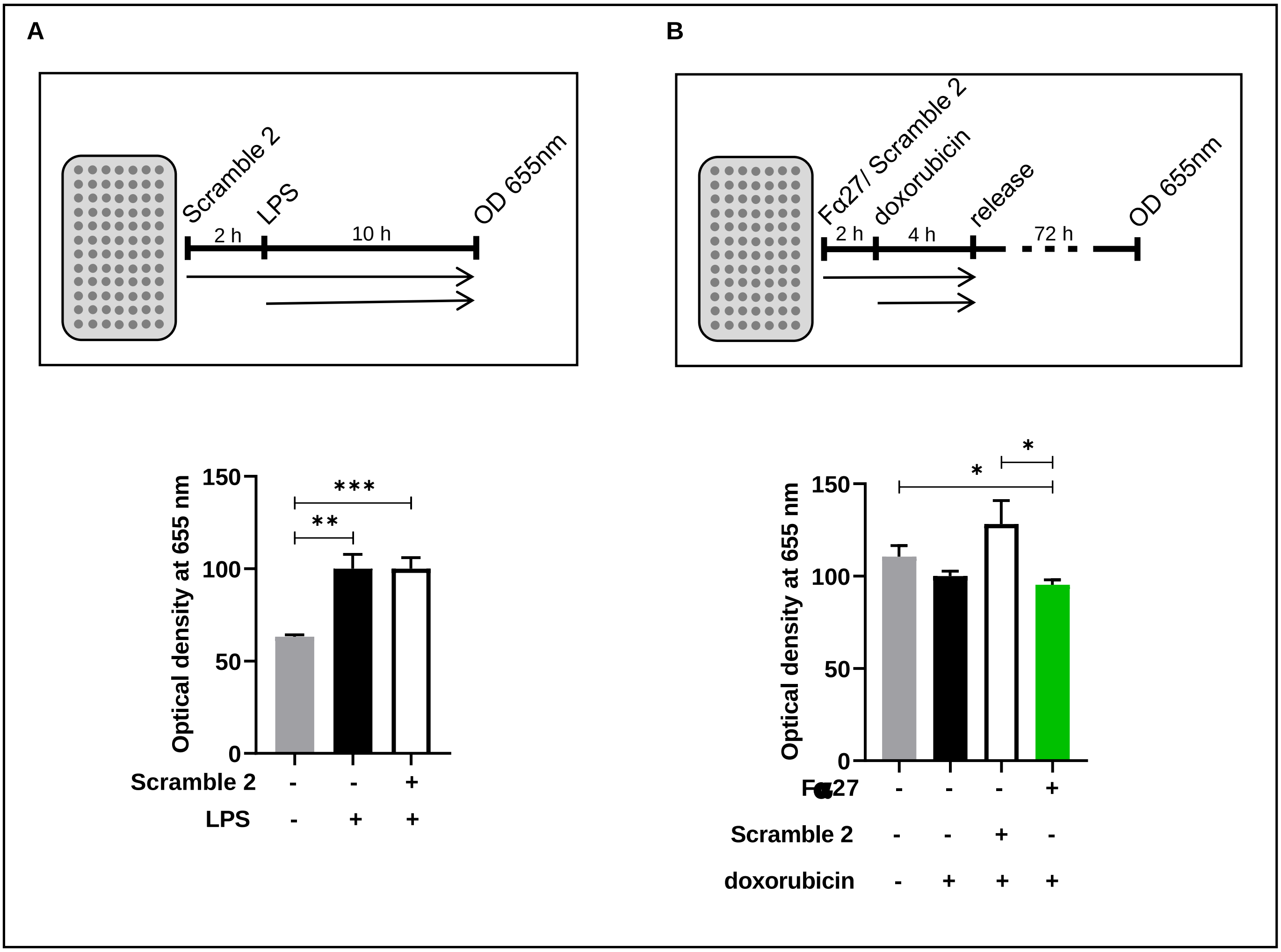

Supplement S6. LPS-induced signaling is not suppressed by Fα27 Scramble 2.

Scheme of experimental analysis of TLR4 signaling and results obtained in HEK-Blue hTLR4 cells pre-treated for 2 h with 100 µg/ml of Scramble 2 peptide followed by additional treatment with 10 ng/ml LPS for 10 h **(A)** or pre-treated with 100 µg/ml Fα27 or Scramble 2 for 2 h followed by additional treatment with 0.16 µM doxorubicin for 4 h, release and cultivation for 72 h **(B)**. LPS treatment **(A)** led to a significant increase of SEAP release detected at OD 655 nm, which was not affected by Scramble 2 pre-treatment. **(B)** Doxorubicin treatment alone did not significantly alter SEAP release, but the comparison of SEAP release after Scramble 2 and Fα27 pre-treatments exhibited a significant decrease by Fα27. Mean OD values of LPS or doxorubicin-treated controls without peptide were set to 100% in each experiment. Data (n=12 obtained in two independent experiments) are shown as mean +SEM (*p<0.05; **p<0.01).

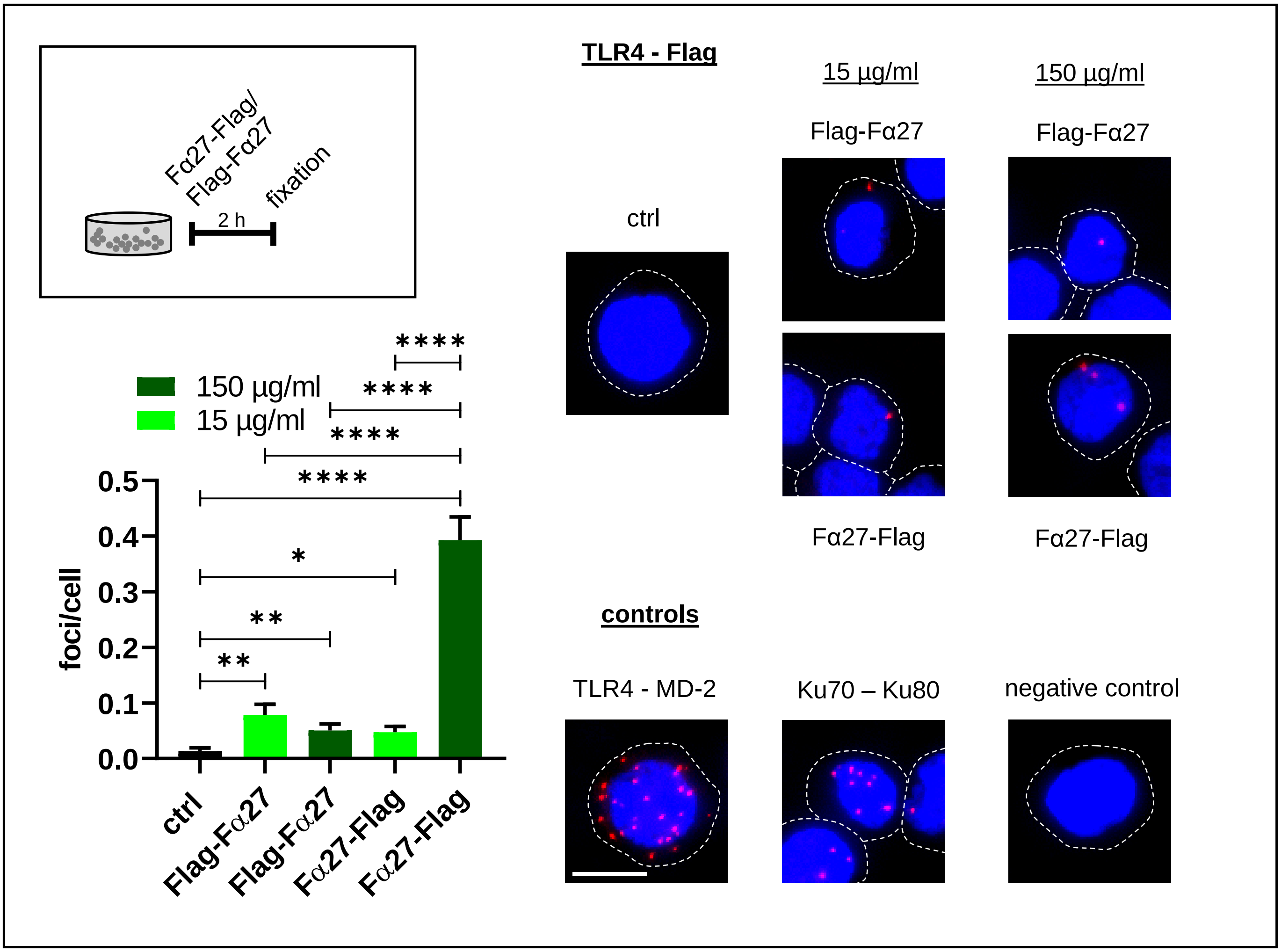

Supplement S7. Fα27 interacts with TLR4 on WTK1 cells.

WTK1 cells were seeded for 2 h with Fα27-Flag, Flag-Fα27 or water (ctrl). A PLA was performed with a Flag- and a TLR4-antibody. The negative control of the PLA was performed without antibodies, positive controls with antibodies for TLR4 binding partner MD-2 and TLR4 and for KU70 and KU80 in the nucleus. The highest foci formation per cells was seen for the highest concentration of Fα27-Flag. Margins of cells are marked by white stippled lines and the scale bar indicates 10 µM. Two independent experiments (n=275-487) represented as mean +SEM (p<0.05; **p<0.01; ****p<0.0001). See also **Figure 7**.
